# Effects of *Lactiplantibacillus plantarum* supplementation on exercise performance and recovery: a systematic review and narrative synthesis

**DOI:** 10.3389/fnut.2026.1881322

**Published:** 2026-07-15

**Authors:** Ji Yanyi, Wang Xingzhi, Daniel H. K. Chow

**Affiliations:** 1 Guangzhou University, Guangzhou, China; 2 Department of Health and Physical Education, The Education University of Hong Kong, Hong Kong, Hong Kong SAR, China; 3 Guangzhou Institute of Sports Science, Guangzhou, China

**Keywords:** exercise performance, fatigue recovery, *Lactiplantibacillus plantarum*, postbiotics, probiotics, strain specificity

## Abstract

**Background:**

*Lactiplantibacillus plantarum* is a prominent probiotic species in sports nutrition; however, its potential ergogenic benefits have not yet been systematically consolidated, particularly regarding strain-specific variations.

**Objective:**

This systematic review aimed to assess the efficacy of *L. plantarum* supplementation on exercise performance and recovery in healthy cohorts, while identifying differential effects across distinct strains.

**Methods:**

Randomized controlled trials (RCTs) were retrieved from PubMed, Web of Science, Scopus, SPORTDiscus, and the Cochrane Library from database inception through March 11, 2026. Risk of bias was assessed using the Cochrane RoB 2 tool. Given the substantial heterogeneity in probiotic strains, dosing regimens, and outcome measures, a narrative synthesis was conducted to integrate the findings.

**Results:**

The efficacy of *L. plantarum* supplementation is highly strain-specific. TWK10 exhibited the highest evidential consistency in evidence, significantly enhancing aerobic endurance and accelerating the clearance of blood lactate and ammonia; notably, its heat-inactivated form (postbiotics) retained these core metabolic benefits. PS128 functioned primarily as a recovery-oriented strain, effectively mitigating exercise-induced muscle damage (EIMD), suppressing systemic inflammation, and preserving neuromuscular efficiency, although its impact on peak performance metrics remained equivocal. PL-02 showed preliminary potential in attenuating markers of muscle damage and inflammation, albeit within a limited evidence base. Conversely, interventions using unspecified *L. plantarum* strains failed to confer demonstrable or consistent benefits. The RoB 2 assessment indicated moderate methodological quality overall, with primary deficiencies identified in allocation concealment and prospective trial registration. Furthermore, the geographical clustering of studies in Taiwan, China, and the reliance on small-to-moderate sample sizes constrain the global generalizability of these findings.

**Conclusion:**

Supplementation with *L. plantarum* can effectively enhance exercise performance and facilitate recovery in healthy individuals, with outcomes fundamentally dictated by strain specificity. Future large-scale, multicenter, and longitudinal RCTs are warranted to fortify the evidence base and confirm these findings across more diverse populations.

**Systematic review registration:**

https://www.crd.york.ac.uk/PROSPERO/myprospero, identifier CRD420261292490.

## 1 Introduction

Probiotics are defined as “live microorganisms that, when administered in adequate amounts, confer a health benefit on the host” (1). These microorganisms exert systemic health-promoting effects primarily by modulating the host’s intestinal microbial architecture (2, 3). Functioning as a “superorgan,” the gut microbiota plays a pivotal role in orchestrating energy metabolism, immune homeostasis, and systemic inflammatory responses via the bidirectional gut-organ axes (4–8). These physiological processes are fundamental determinants of exercise performance, fatigue recovery, and long-term training adaptations. Under the physiological stress of high-intensity or prolonged exercise, individuals frequently encounter homeostatic perturbations; these include impaired intestinal barrier integrity (often termed “leaky gut”) (9–11), exacerbated systemic inflammation (12, 13), and intensified oxidative stress (14–16). Collectively, these factors represent critical biological constraints that can impede physical recovery and the optimization of athletic performance.

In response to these challenges, probiotic intervention has emerged as a compelling nutritional strategy within exercise science, aimed at fortifying the intestinal microenvironment (17–20). L. plantarum (formerly Lactobacillus plantarum) stands out as a highly representative species due to its robust gastrointestinal resilience, superior colonization capacity, and versatile metabolic repertoire (21, 22). Accumulating evidence from both preclinical and clinical trials indicates that *L. plantarum* not only mitigates gastrointestinal distress but also exhibits significant potential in optimizing glucose and lipid metabolism, attenuating oxidative damage, and facilitating recovery from exercise-induced fatigue (23–28). These multifaceted biological properties suggest that *L. plantarum* may enhance training adaptations through diverse pathways, including improving energy substrate utilization and neutralizing reactive oxygen species (ROS)-mediated cellular injury.

Despite this promising outlook, human-centric research investigating the direct ergogenic effects of *L. plantarum* remains in its relative infancy, and the current evidence base is characterized by significant fragmentation. Existing randomized controlled trials (RCTs) display substantial methodological heterogeneity regarding study cohorts (elite vs. recreational), dosing regimens (strain selection, concentration, and duration), and outcome measures (aerobic endurance vs. muscular power). While several studies report that *L. plantarum* significantly prolongs time-to-exhaustion (TTE), augments muscular strength, and downregulates inflammatory biomarkers (29–35), others have failed to observe statistically significant improvements (36, 37). Such discrepancies create considerable ambiguity for practitioners, including coaches and sports nutritionists, regarding the efficacy of probiotic interventions.

These inconsistencies likely arise from three primary factors. First, the physiological efficacy of probiotics is fundamentally strain-specific, as distinct *L. plantarum* strains possess divergent genomic profiles and biological functionalities (38–40). Second, the ergogenic impact of probiotics may depend upon specific participant characteristics or exercise modalities (41, 42). Third, methodological constraints—such as underpowered sample sizes, suboptimal control designs, and inconsistent endpoint parameters—further compromise the reliability of extant findings (17, 42). Crucially, prior systematic reviews have often aggregated data across heterogeneous genera and species, yielding broad conclusions that fail to address the specific nuances of strain selection, optimal duration, and target demographics.

**FIGURE 1:**
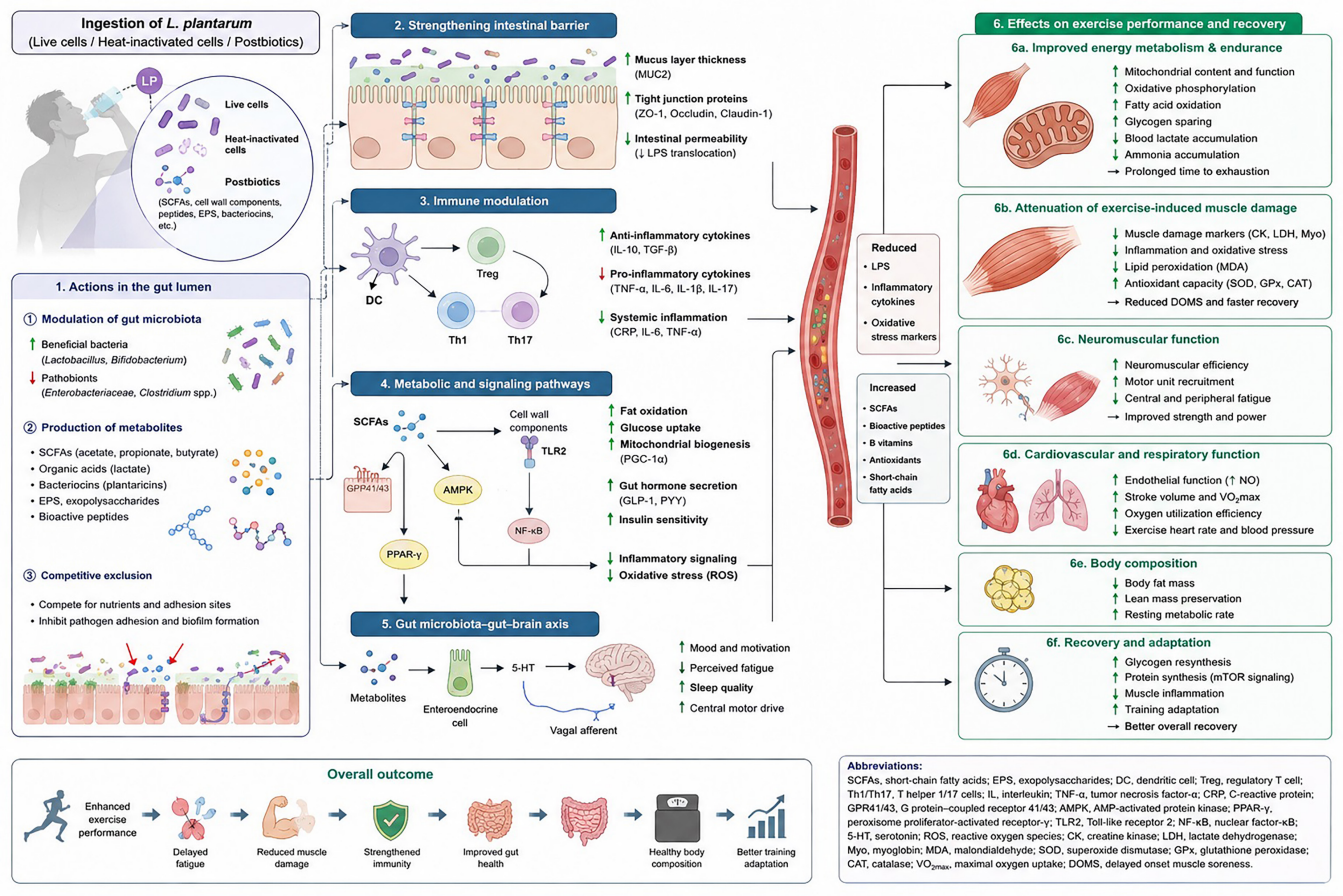
Biological mechanisms of *Lactiplantibacillus plantarum* in enhancing exercise performance and recovery.

Consequently, there is a compelling need for a systematic review anchored in strain specificity that focuses exclusively on *L. plantarum* in healthy populations. By integrating a comprehensive spectrum of exercise-related outcomes, this study seeks to elucidate the differential efficacy across distinct strains and formulations, thereby bridging a critical gap in the literature. These findings aim to provide evidence-based guidance for sports nutrition practice and establish a robust scientific foundation for gut microbiota-targeted nutritional strategies designed to optimize athletic excellence (Figure 1).

## 2 Methods

### 2.1 Protocol and registration

This systematic review was prospectively registered with the International Prospective Register of Systematic Reviews (PROSPERO) on January 24, 2026 (Registration No.: CRD420261292490). The study was conducted and reported in strict adherence to the Preferred Reporting Items for Systematic Reviews and Meta-Analyses (PRISMA 2020) statement. Several operational definitions were established for the inclusion criteria to ensure methodological reproducibility without compromising the core eligibility framework.

### 2.2 Search strategy

A comprehensive literature search was executed from database inception through March 11, 2026, across seven electronic databases: PubMed, the Cochrane Library, Web of Science, Scopus, SPORTDiscus, CNKI, and Wanfang Data. The search strategy employed a combination of Medical Subject Headings (MeSH) and relevant keywords centered on four thematic pillars: (1) *Lactiplantibacillus plantarum*; (2) healthy cohorts; (3) outcomes concerning exercise performance and physiological recovery; and (4) randomized controlled trials (RCTs). The detailed search algorithms for each database are provided in Supplementary Material S1.

### 2.3 Inclusion and exclusion criteria

Eligibility criteria were systematically defined using the PICOS (Participants, Interventions, Comparators, Outcomes, and Study Design) framework. A concise overview of the eligibility criteria is summarized in Table 1

**TABLE 1:** Simplified PICOS framework for study eligibility.

PICOS component	Inclusion criteria	Exclusion criteria
Population	Healthy adults (sedentary, recreational, or elite athletes)	Clinical, diseased, or hospitalized populations
Intervention	Oral *L. plantarum* (viable or postbiotics; single or multi-strain)	Studies unable to isolate *L. plantarum* effects
Comparison	Placebo or no-supplementation controls	Studies lacking a proper control group
Outcomes	Exercise performance markers, fatigue recovery, muscle damage indicators, inflammatory biometrics	Studies lacking objective physical/physiological outcomes
Study Design	Randomized controlled trials (RCTs)	Animal studies, case reports, and non-RCT reviews

#### 2.3.1 Participants

Healthy adults aged ≥ 18 years, defined as individuals without a diagnosed history of chronic, metabolic, or cardiovascular pathologies, as confirmed by screening protocols in the original studies. Eligible cohorts included: (i) sedentary individuals (defined by regular physical activity < 1 session/week or failing to meet World Health Organization (43) activity guidelines); (ii) recreational exercisers; and (iii) elite or sub-elite athletes. Clinical or hospitalized populations were excluded, while community-dwelling older adults with CFS scores of 1–4 were deemed eligible.”

#### 2.3.2 Interventions

Oral administration of any *L. plantarum* strain, encompassing both viable probiotics and heat-inactivated preparations (postbiotics), with no restrictions on dosage or intervention duration. Only single-strain interventions or well-characterized multi-strain products were eligible. Studies involving “probiotic cocktails” with ambiguous compositions where the specific contribution of *L. plantarum* could not be isolated were excluded.

#### 2.3.3 Comparators

Matched placebos devoid of active ingredients known to modulate metabolic or physical performance.

#### 2.3.4 Outcomes

At least one objective quantification of exercise performance, muscular function, or post-exercise recovery kinetics. Trials focused primarily on clinical disease markers were excluded.

#### 2.3.5 Study design

Peer-reviewed RCTs utilizing parallel-group or crossover designs. Acute, single-dose intervention studies were excluded to focus on chronic adaptation.

Language and Publication Type: Only full-text articles published in English or Chinese in peer-reviewed journals were considered.

### 2.4 Study selection and data extraction

Literature screening was performed independently by two reviewers. Following the removal of duplicate records, titles and abstracts were screened against the eligibility criteria, followed by a rigorous full-text appraisal. Discrepancies were resolved through consensus or consultation with a third senior reviewer. A standardized form was used for data extraction, covering: bibliographic details, strain identification, study design, cohort demographics (sample size and sex distribution), intervention parameters (dose, formulation, and duration), primary/secondary outcomes, and safety profiles. While a quantitative meta-analysis was initially considered, it was ultimately rejected due to extensive clinical, methodological, and statistical heterogeneity (e.g., variations in *L. plantarum* strains, training status, and non-comparable outcome modalities like time-to-exhaustion vs. maximal contractions). Exploratory subgroup analyses of selected outcomes were unfeasible as the resulting strata yielded fewer than three independent trials per sub-category, lacking adequate statistical power. Consequently, a structured narrative synthesis utilizing systematic summaries and categorical comparisons was adopted. For primary studies wherein descriptive statistics were omitted but retrospective inferential statistics from independent two-sample designs were available, standardized effect sizes (Cohen’s *d*) were mathematically estimated to enhance comparability. The *p*-value was converted to its corresponding critical t-statistic (df=n_1_+n_2_−2) via the inverse cumulative distribution function, and Cohen’s *d* was derived as:$d=t\times \sqrt{1/n_{2}+1/n_{2}}$ (44, 45).

### 2.5 Risk of bias assessment

Methodological quality was appraised using the Cochrane Risk of Bias 2 (RoB 2) tool (46). Two reviewers independently evaluated five domains: (i) the randomization process; (ii) deviations from intended interventions; (iii) missing outcome data; (iv) measurement of the outcome; and (v) selection of the reported result. Each domain, as well as the overall risk, was categorized as “Low risk,” “High risk,” or “Some concerns.” Disagreements were adjudicated by a third reviewer.

### 2.6 Outcome measures

The primary outcome was exercise performance, categorized into:

*Aerobic endurance:* e.g., Time to Exhaustion (TTE), maximal oxygen uptake (VO_2m*ax*_), and Yo-Yo test performance.
*Anaerobic capacity*: e.g., Wingate and RAST test metrics.
*Muscular function:* e.g., Handgrip strength, Maximal Voluntary Isometric Contraction (MVIC), Countermovement Jump (CMJ) height, Rate of Force Development (RFD), Relative Peak Force (RPF), Peak Rate of Force Development (pRFD)

Secondary outcomes focused on recovery kinetics, including:

*Muscle damage markers:* Creatine Kinase (CK) and Myoglobin.
*Inflammatory biomarkers:* C-reactive protein (CRP), IL-6, TNF-α, and IL-10.

Metabolic recovery: Blood lactate, ammonia, and glucose levels.

*Neuromuscular function*: Median Frequency (MDF), integrated EMG (iEMG), and Neuromuscular Efficiency (NME).
*Exploratory outcomes*: included gut microbiota profiling (α/β diversity and taxonomic abundance) and Short-Chain Fatty Acid (SCFA) concentrations (acetate, propionate, and butyrate).

## 3 Results

### 3.1 Study selection process

**FIGURE 2:**
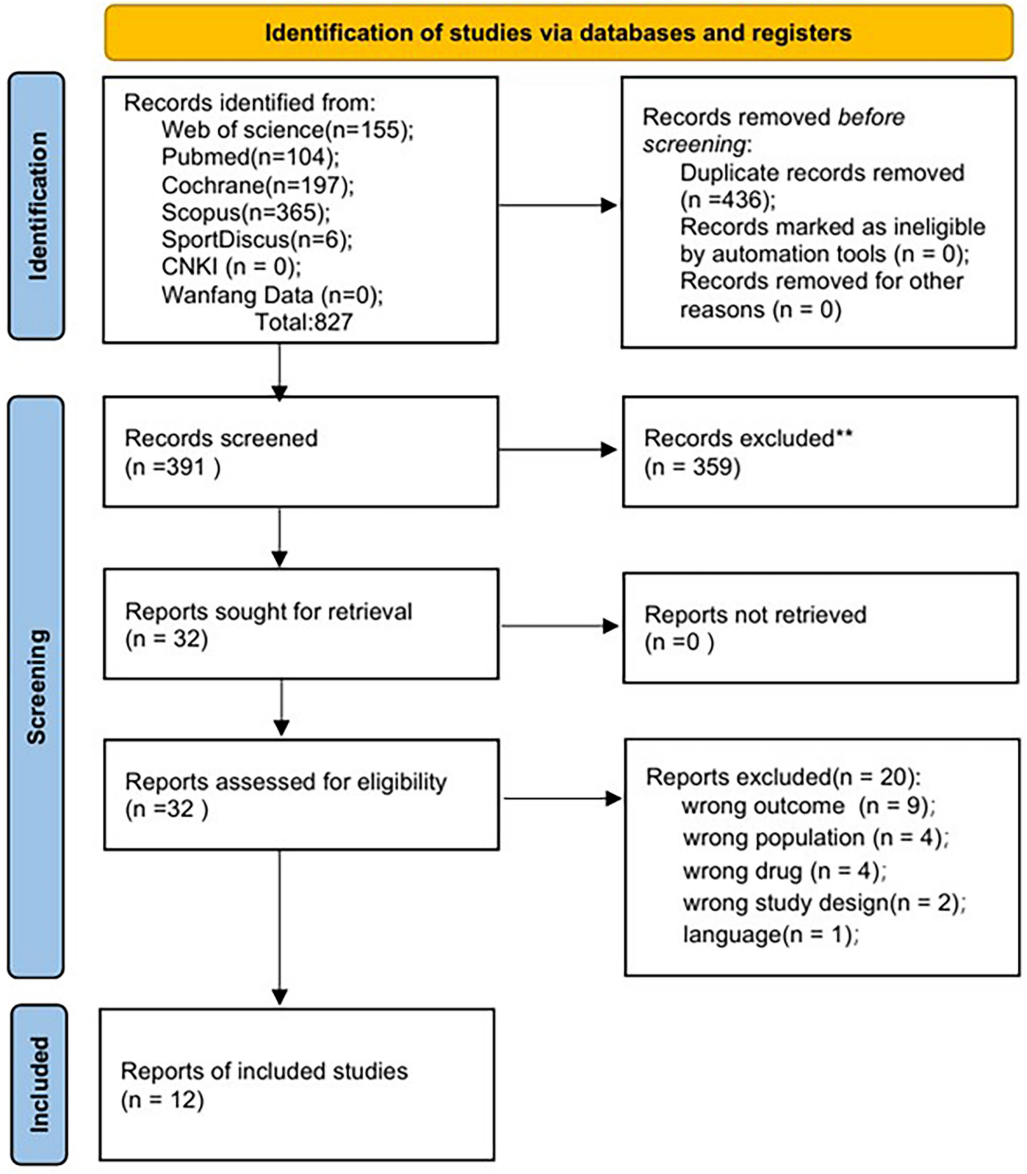
PRISMA flow diagram.

The initial systematic search across seven electronic databases yielded a total of 827 records: Web of Science (*n* = 155), PubMed (*n* = 104), the Cochrane Library (*n* = 197), Scopus (*n* = 365), SPORTDiscus (*n* = 6), CNKI (*n* = 0), and Wanfang Data (*n* = 0). Following the removal of duplicate records, 391 unique citations remained for preliminary screening. During the title and abstract screening phase, 359 records were excluded for failing to meet the predefined eligibility criteria, leaving 32 candidates for comprehensive full-text appraisal. Upon rigorous secondary assessment, 20 additional studies were excluded based on the following justifications: (i) inappropriate outcome measures (*n* = 9); (ii) ineligible study populations (*n* = 4); (iii) non-compliant intervention protocols (*n* = 4); (iv) inappropriate study designs (*n* = 2); and (v) unacceptable language/publication types (*n* = 1). Ultimately, 12 randomized controlled trials (RCTs) met all inclusion criteria and were synthesized in this systematic review. The detailed PRISMA selection flow is illustrated in Figure 2.

### 3.2 Study characteristics

A total of 12 randomized controlled trials (RCTs) were included in this systematic review, all of which employed a double-blind, placebo-controlled framework, including two crossover designs. The aggregate sample size comprised 446 participants, with ages ranging from 18 to 85 years. The demographic profile was highly diverse, encompassing healthy sedentary adults, recreational exercisers, amateur athletes, and mildly frail elderly individuals. Regarding the intervention protocols, the specific strains investigated included *L. plantarum* TWK10 (*n* = 6 trials, including one evaluating a heat-inactivated postbiotic preparation), PS128 (*n* = 4), PL-02 (*n* = 1), and one trial involving an unspecified strain. Daily dosages varied significantly, ranging from 1 × 10^9^ to 3 × 10^11^ CFU. Intervention durations spanned from 3 to 18 weeks, with the majority (10/12 trials) concentrated between 4 and 6 weeks. Notably, no serious adverse events or significant intolerance were reported across all included studies. Detailed characteristics of the individual studies are synthesized in Table 2.

**TABLE 2:** Characteristics of the included studies.

Study	Strain	Study design	Sample size	Population	Intervention dose	Duration	Primary outcome measure
Huang et al. (25)	TWK10	RCT, double-blind, parallel	54	Healthy adults	3 × 10^10^/9 × 10^10^ CFU/day	6 weeks	TTE, lactate, ammonia, glucose, NLR, PLR
Lee et al. (33)	TWK10	RCT, double-blind, parallel	53	Healthy adults	3 × 10^11^ CFU/day (live/heat-killed)	6 weeks	TTE, lactate, ammonia, glucose, CK, NLR, PLR, gut microbiota, SCFAs
Lee et al. (34)	TWK10	RCT, double-blind, parallel	55	Frail elderly	2 × 10^10^/6 × 10^10^ CFU/day	18 weeks	handgrip strength, functional performance
Huang et al. (30)	TWK10	RCT, double-blind, parallel	16	Healthy males	1 × 10^11^ CFU/day	6 weeks	TTE, VO_2_max, lactate, ammonia, glucose, FFA
Chen et al. (36)	TWK10	RCT, double-blind, parallel	47	Obese females	6 × 10^10^ CFU/day	8 weeks	TTE, VO2max, metabolic parameters
Cheng et al. (29)	TWK10-HK	RCT, double-blind, parallel	30	Healthy males	3 × 10^10^ cells/day	6 weeks	TTE, handgrip strength, lactate, ammonia, glucose,
Fu et al. (37)	PS128	RCT, double-blind, crossover	8	Recreational runners	3 × 10^10^ CFU/day	4 weeks	VO_2_max, Wingate, Knee extensor, flexor MVIC, CK, LDH, SOD, CAT, BCAA, NH_3_
Yu et al. (35)	PS128	RCT, double-blind, crossover	8	Recreational runners	3 × 10^10^ CFU/day	4 weeks	MDF, iEMG, NME, Knee extensor MVIC
Huang et al. (31)	PS128	RCT, double-blind, parallel	34	Triathletes	3 × 10^10^ CFU/day	3–4 weeks	85%VO_2_max, Wingate, lactate, ammonia, FFA, inflammatory markers
Huang et al. (32)	PS128	RCT, double-blind, parallel	20	Triathletes	3 × 10^10^ CFU/day	4 weeks	TTE, VO_2_max, NLR, gut microbiota, SCFAs
Lee et al. (47)	PL-02	RCT, double-blind, parallel	88	Healthy adults	1.5 × 10^10^ CFU/day	6 weeks	VO2max, CK, IMTP-RPF, IMTP-pRFD, CMJ-RFD, CMJ-RPF, inflammatory markers, gut microbiota
Santibañez-Gutierrez et al. (48)	Unspecified	RCT, double-blind, parallel	22	CrossFit athletes	1 × 10^10^ CFU/day	4 weeks	YOYO IR1 test, CMJ

TTE, Time to Exhaustion; FFA, Free Fatty Acids; MVIC, Maximal Voluntary Isometric Contraction; MDF, Mean Displacement Frequency; iEMG, Integrated Electromyography; NME, Neuromuscular Efficiency; IMTP, Isometric Mid-Thigh Pull; RPF, Relative Peak Force; pRFD, Peak Rate of Force Development; CMJ, Countermovement Jump; RFD, Rate of Force Development; YOYO IR1, Yo-Yo Intermittent Recovery Test Level 1; TWK10-HK, TWK10 heat-killed.

### 3.3 Risk of bias assessment

**FIGURE 3:**
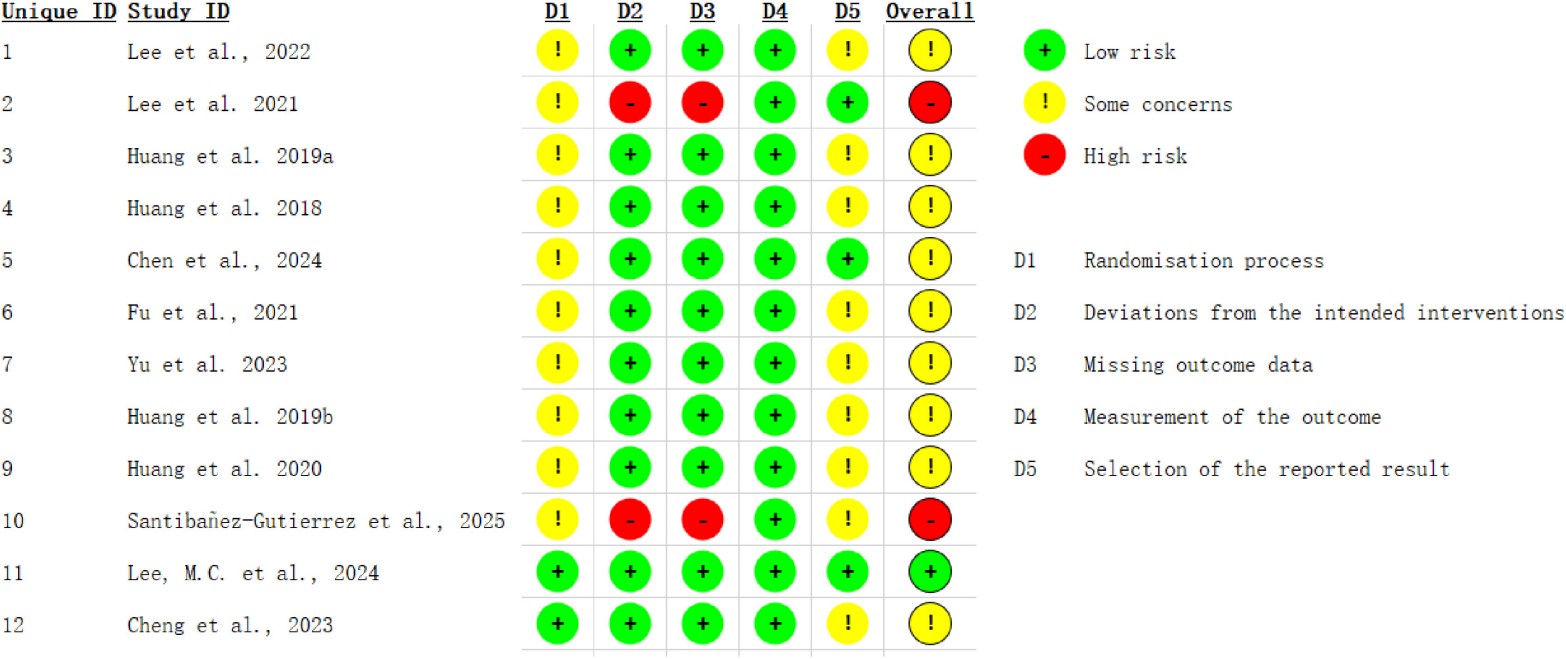
Risk of bias in individual studies.

**FIGURE 4:**
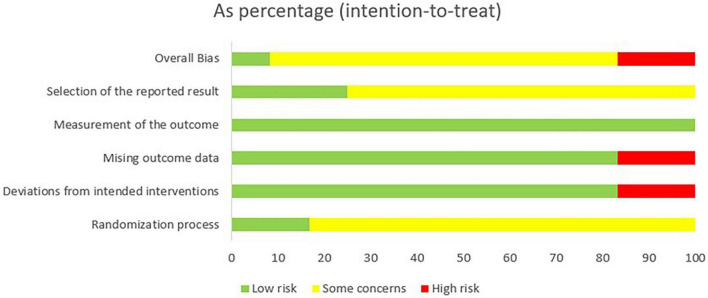
Risk of bias summary.

Methodological quality was rigorously appraised using the Cochrane Risk of Bias 2 (RoB 2) tool (Figures 3, 4). The overall evidentiary quality of the included trials was categorized as moderate.

- *Randomization process:* Only 2 trials were rated as “Low risk,” while 10 trials raised “Some concerns,” primarily attributed to insufficient reporting of allocation concealment procedures.
- *Deviations from intended interventions and missing outcome data*: Ten trials (83.3%) achieved a “Low risk” rating. Conversely, Lee et al. (49) and Santibañez-Gutierrez et al. (48) were classified as “High risk” due to substantial attrition rates (23.6 and 33.3%, respectively) and the omission of Intention-to-Treat (ITT) analyses.
- *Measurement of the outcome*: This domain exhibited the highest degree of rigor, with all trials categorized as “Low risk” due to the implementation of objective and standardized assessment protocols.
- *Selection of the reported result:* Three trials were judged to be at “Low risk” based on accessible prospective registrations. Eight trials raised “Some concerns” due to the absence of public protocol registration, while one trial was deemed “High risk” owing to discrepancies between the registered protocol and the published findings.

In synthesis, the collective risk of bias across the evidence base is acceptable, yielding a moderate level of reliability. The primary methodological caveats identified include a lack of detailed allocation concealment (observed in 83% of trials) and a suboptimal prospective registration rate (33%), both of which warrant cautious interpretation of the findings.

### 3.4 Exercise performance

#### 3.4.1 Aerobic endurance aerobic

Endurance represented the most frequently evaluated exercise performance metric in this review. A total of 10 studies quantified aerobic capacity utilizing Time to Exhaustion (TTE), maximal oxygen uptake (VO_2m*ax*_), and the Yo-Yo Intermittent Recovery Level 1 (Yo-Yo IR1) test. These trials investigated *L. plantarum* strains TWK10 (*n* = 5), PS128 (*n* = 3), PL-02 (*n* = 1), and unspecified strains (*n* = 1). The modulation of aerobic endurance demonstrated pronounced strain specificity (Table 3).

**TABLE 3:** Summary of study characteristics and effects of different *Lactiplantibacillus plantarum* strains on aerobic endurance outcomes.

Strain	Study (year)	Population	Intervention	Outcome	Main result	Effect size [Cohen’s *d* (95% CI)]	*p*-value
TWK10	Lee et al. (33)	Healthy adults (*n* = 53)	Viable: 3 × 10^11^ CFU, 6wk	TTE	↑ vs. placebo	1.69 (0.91, 2.47)	*P* < 0.001
	Huang et al. (25)	Healthy adults (*n* = 54)	HK: 3 × 10^11^ cells, 6wk	TTE	↑ vs. placebo	1.01 (0.32, 1.71)	*P* < 0.01
			Low: 3 × 10^10^ CFU, 6wk	TTE	↑ vs. placebo	(NR)	*p* = 0.0207
	Huang et al. (30)	Healthy males (*n* = 16)	High: 9 × 10^10^ CFU, 6wk	TTE	↑ vs. placebo	(NR)	*p* < 0.001
			High: 9 × 10^10^ CFU, 6wk	TTE	↑ vs. low dose	(NR)	*p* = 0.0336
			1 × 10^11^ CFU, 6 wk	TTE	↑1.58-fold vs. placebo	3.07 (1.57, 4.57)	*P* = 0.04
	Chen et al. (36)	Obese females (*n* = 47)	6 × 10^10^ CFU, 8 wk	TTE/VO2max	↔ vs. placebo	(NR)	*p* > 0.05
	Cheng et al. (29)	Healthy males (*n* = 30)	HK: 3 × 10^10^ cells, 6 wk	TTE	↑1.24-fold vs. placebo	(NR)	*p* = 0.0028
PS128	Fu et al. (37)	Recreational runners (*n* = 8, half-marathon)	3 × 10^10^ CFU, 4 wk	VO2max	↔ vs. placebo	(NR)	*p* > 0.05
	Huang et al. (32)	Triathletes (*n* = 20)	3 × 10^10^ CFU, 4 wk	TTE	↑130% vs. placebo	(NR)	*p* = 0.0035
	Huang et al. (31)	Triathletes (*n* = 16)	3 × 10^10^ CFU, 3 wk	VO2max	↔ vs. placebo	(NR)	*p* > 0.05
				TTE	↑ vs. placebo	3.09 (1.64, 4.54)	*P* < 0.05
PL-02	Lee et al. (47)	Healthy adults (*n* = 88)	1.5 × 10^10^ CFU, 6 wk	VO2max	↔ vs. placebo	0.41 (−0.19, 1.01)	*p* = 0.018
Unspecified *L. plantarum*	Santibañez-Gutierrez et al. (48)	Recreational CrossFit athletes (*n* = 28)	1 × 10^10^ CFU, 4 wk	YOYO IR1 test	↔ vs. placebo	0.26 (−0.59, 1.10)	*p* > 0.05

↑, significant increase; ↔, no significant change; HK, heat-killed; TTE, time to exhaustion; VO2max, maximal oxygen consumption; Yo-Yo IR1 test, Yo-Yo Intermittent Recovery Test Level 1;CFU, colony-forming units (viable bacteria count); cells, total bacterial cells (including heat-killed forms); wk, week(s); CI, confidence interval; NR, not reported (insufficient data to calculate effect size and 95% CI).

^1^ Effect sizes marked with

^1^ were estimated from reported *P*-values and sample sizes due to missing descriptive statistics; unmarked effect sizes were calculated from original means and SDs Cohen’s *d*: small (0.2), medium (0.5), large (0.8).

TWK10 demonstrated relatively consistent preliminary evidence for supporting aerobic endurance. Four out of five trials reported significant ergogenic improvements, with TTE increasing by 1.24–1.58-fold compared to placebo cohorts, corresponding to substantial effect sizes (Cohen’s *d* = 1.01–3.07). Dose-response analyses indicated that high-dose supplementation (9 × 10^10^CFU/day) was significantly superior to low-dose regimens (3 × 10^10^CFU/day), establishing an effective therapeutic threshold of at least 3 × 10^10^ CFU/day over a 6-week intervention period. Notably, the ergogenic efficacy of heat-inactivated TWK10 (postbiotics) was comparable to that of its viable counterpart. The sole null finding occurred in a cohort of middle-aged obese females, suggesting that adiposity status and biological sex may serve as critical effect modifiers in probiotic-mediated endurance enhancement.

The impact of PS128 on aerobic endurance remained equivocal and appeared contingent upon both the target population and the specific metrics employed. In competitive triathletes, supplementation (3 × 10^10^CFU/day for 3–4 weeks) significantly prolonged TTE, despite negligible changes in VO_2m*ax*_. Conversely, no discernible improvements in VO_2m*ax*_ were observed in recreational runners at the same dosage. All PS128 trials utilized a fixed-dose design, thereby precluding a definitive dose-response characterization.

Regarding PL-02, a 6-week intervention (CFU/day) failed to significantly elevate, although its influence on TTE has yet to be elucidated. In contrast, unspecified *L. plantarum* strains failed to confer any significant benefits across all aerobic endurance parameters.

In summary, the ergogenic benefits of *L. plantarum* on aerobic capacity appear to be highly strain-specific. Based on the available exploratory data, TWK10 shows the most consistent trend, suggesting dose-dependent enhancements in TTE that are largely preserved in postbiotic formulations. Conversely, The effects of PS128 are inconsistent, with benefits primarily restricted to TTE in elite athletic cohorts rather than recreational populations. Regarding PL-02 and unspecified strains, neither has conferred significant improvements across aerobic endurance parameters within the currently limited literature.

#### 3.4.2 Anaerobic capacity

A total of four studies evaluated the impact of *L. plantarum* on anaerobic capacity, utilizing the Wingate anaerobic test (*n* = 3) and the Running Anaerobic Sprint Test (RAST, *n* = 1). These investigations focused on *L. plantarum* PS128 (*n* = 2), PL-02 (*n* = 1), and unspecified strains (*n* = 1); notably, no evaluations of anaerobic performance were identified for the TWK10 strain. The ergogenic effects across different strains exhibited substantial inconsistency, underpinned by an overall sparse evidence base (Table 4).

**TABLE 4:** Summary of study characteristics and effects of different *Lactiplantibacillus plantarum*strain on anaerobic capacity outcomes.

Strain	Study (year)	Population	Intervention	Outcome	Main result	Effect size [Cohen’s *d* (95% CI)]	*p*-value
PS128	Huang et al. (31)	Triathletes (*n* = 16)	3 × 10^10^ CFU, 3 wk	Wingate-PP(W)	↑ vs. placebo	2.62 (1.28, 3.96)	*p* < 0.05
				Wingate-MP(W)	↑ vs. placebo	3.43 (1.89,4.97)	*p* < 0.05
				Wingate-FI(%)	↓ vs. placebo	−2.99 (−4.42, −1.56)	*p* < 0.05
PS128	Fu et al. (37)	Recreational runners (*n* = 8)	3 × 10^10^ CFU, 4 wk	Wingate-PP(W)	↔	(NR)	p > 0.05
				Wingate-MP(W)	↔	(NR)	p > 0.05
				Wingate-FI(%)	↔	(NR)	p > 0.05
PL-02	Lee et al. (47)	Healthy non-athletes (*n* = 88)	1.5 × 10^10^ CFU/d, 6 wk	Wingate-PP(W)	↔	0.24 (−0.36, 0.83)	*p* > 0.05
				Wingate-MP(W)	↔	0.20 (−0.39, 0.79)	*p* > 0.05
Unspecified *L. plantarum*	Santibañez-Gutierrez et al. (48)	Recreational CrossFit athletes (*n* = 22)	1 × 10^10^ CFU, 4 wk	RAST (35 m sprint time)	↔	0.167 (−0.678, 1.012)	*p* > 0.05

↑, significant increase; ↓, significant decrease; ↔, no significant change; PP, peak power; MP, mean power; FI, fatigue index; RAST, Running-Based Anaerobic Sprint Test; CFU, colony-forming units; wk, week(s); CI, confidence interval; NR, not reported (insufficient data to calculate effect size and 95% CI).

^1^Effect sizes marked with

^1^ were estimated from reported *P*-values and sample sizes due to missing descriptive statistics; unmarked effect sizes were calculated from original means and SDs Cohen’s *d*: small (0.2), medium (0.5), large (0.8).

The efficacy of PS128 appeared to be highly population-dependent. In competitive triathletes, a 3-week intervention (3 × 10^10^ CFU/day) elicited profound improvements in Wingate peak power, mean power, and fatigue index, with substantial effect sizes of 2.62, 3.43, and −2.99, respectively. Conversely, the identical dosage yielded no statistically significant benefits in recreational runners, suggesting that the anaerobic ergogenic potential of PS128 may be modulated by the baseline training status of the cohort. All PS128-related trials employed a fixed-dose design, which precluded the characterization of a dose-response relationship.

Regarding PL-02, a 6-week supplementation regimen (1.5 × 10^10^ CFU/day) failed to produce significant enhancements in either Wingate peak or mean power (*d* = 0.24 and 0.20, respectively). Similarly, unspecified *L. plantarum* strains demonstrated no discernible influence on RAST sprint performance.

In summary, the evidentiary support for *L. plantarum* in augmenting anaerobic capacity remains nascent. PS128 appears to confer ergogenic benefits exclusively in elite athletic populations, while remaining ineffective in recreational cohorts. Neither PL-02 nor unspecified strains have demonstrated consistent anaerobic advantages to date. Furthermore, the potential impact of TWK10 on anaerobic performance remains a critical knowledge gap in the current literature.

#### 3.4.3 Muscular strength

Outcomes evaluating muscular strength and explosive performance encompassed Maximal Voluntary Isometric Contraction (MVIC), Relative Peak Force during Isometric Mid-Thigh Pull (IMTP-RPF), Rate of Force Development (IMTP-pRFD and CMJ-RFD), Countermovement Jump Relative Peak Force (CMJ-RPF), jump height (CMJ height), and handgrip strength. A total of six studies reported these strength-related metrics. The ergogenic impact of distinct *L. plantarum* strains on muscular performance demonstrated distinct strain specificity, with dose-response relationships varying according to specific functional outcomes (Table 5).

**TABLE 5:** Summary of study characteristics and effects of different *Lactiplantibacillus plantarum* strains on muscle strength outcomes.

Strain	Study (year)	Population	Intervention	Outcome	Main result	Effect size [Cohen’s *d* (95% CI)]	*p*-value
TWK10	Lee et al. (50)	Frail older adults (*n* = 55)	2 × 10^10^ CFU/d, 18 wk	Left handgrip strength	↔ vs. placebo	(NR)	*p* > 0.05
			6 × 10^10^ CFU/d, 18 wk	Right handgrip strength	↔ vs. placebo	(NR)	*p* > 0.05
TWK10-HK	Cheng et al. (29)	Healthy males (*n* = 30, 20–40 y, untrained)	3 × 10^10^ cells/d, 6 wk	Left handgrip strength	↑ vs. placebo	(NR)	*P* = 0.0140
				Right handgrip strength	↑ vs. placebo	(NR)	*P* = 0.0002
PS128	Fu et al. (37)	Recreational runners (*n* = 8, half-marathon)	3 × 10^10^ CFU/d, 4 wk	Knee extensor MVIC	↑ vs. placebo (3–96 h post-HM)	2.09 (0.87, 3.31)^1^ (48 h post-HM)	*p* < 0.0001 (24 h)
				Knee flexor MVIC	↑ vs. placebo (24 h post-HM)	1.45 (0.33, 2.57)^1^	*P* = 0.0116
				CMJ height	PS128 group maintained power; placebo group significantly decreased	(NR)	< 0.05 (placebo vs. baseline)
PS128	Yu et al. (35)	Recreational runners (*n* = 8, half-marathon)	3 × 10^10^ CFU/d, 4 wk	Knee extensor MVIC	↑ vs. placebo (0–96 h post-HM)	2.07 (0.86, 3.28)^1^ (24 h post-HM)	< 0.0001 (24 h)
PL-02	Lee et al. (47)	Healthy non-athletes (*n* = 88, 20–40 y)	1.5 × 10^10^ CFU/d, 6 wk	IMTP-RPF	↔ vs. placebo	0.13 (−0.46, 0.72)	*p* > 0.05
				IMTP-pRFD	↔ vs. placebo	0.23 (−0.36, 0.82)	*p* > 0.05
				CMJ-RFD	↔ vs. placebo	0.06 (−0.53, 0.65)	*p* > 0.05
				CMJ-RPF	↔ vs placebo	0.33 (−0.27, 0.92)	*p* > 0.05
Unspecified *L. plantarum*	Santibañez-Gutierrez et al. (48)	Recreational CrossFit athletes (*n* = 22)	1 × 10^10^ CFU/d, 4 wk	CMJ height	↔ vs. placebo	0.18 (−0.66, 1.02)	*p* > 0.05

↑, significant increase; ↓, significant decrease; ↔, no significant change; HK, heat-killed; CFU, colony-forming units (viable bacteria count); cells, total bacterial cells (including heat-killed forms); HM, half-marathon; MVIC, maximum voluntary isometric contraction; CMJ, countermovement jump; IMTP, isometric mid-thigh pull; pRFD, peak rate of force development; RFD, rate of force development; RPF, relative peak force; CI, confidence interval; NR, not reported (insufficient data to calculate effect size and 95% CI).

^1^ Effect sizes marked with

^1^ were estimated from reported *P*-values and sample sizes due to missing descriptive statistics; unmarked effect sizes were calculated from original means and SDs Cohen’s *d*: small (0.2), medium (0.5), large (0.8).

PS128 provided the most consistent evidentiary support for attenuating post-exercise decrements in neuromuscular function. In recreational runners, supplementation with PS128 (3 × 10^10^CFU/day for 4 weeks) effectively preserved knee extensor and flexor strength following a half-marathon, yielding substantial effect sizes (Cohen’s *d* = 2.09 and 1.45, respectively), and mitigated the reduction in CMJ height. An independent trial further validated its protective efficacy on extensor torque (*d* = 2.07). Although all PS128 trials employed a fixed-dose design—precluding a definitive dose-response characterization—the protective efficacy remained highly consistent across the 4-week intervention period.

The effects of TWK10 (in both viable and heat-inactivated forms) on upper-body strength were modulated by cohort characteristics and formulation modalities. Heat-inactivated TWK10 (3 × 10^10^ cells/day for 6 weeks) significantly enhanced handgrip strength in healthy males. Conversely, high-dose viable TWK10 (6 × 10^10^CFU/day for 18 weeks) elicited no statistically significant between-group differences in frail elderly individuals. This divergence in findings, compounded by variations in population demographics, formulation types, and intervention timelines, precludes definitive conclusions regarding the dose-response or time-course efficacy of TWK10 for strength enhancement.

Regarding PL-02 (1.5 × 10^10^CFU/day for 6 weeks), although significant intra-group improvements in strength-related kinetic variables were observed relative to baseline, the magnitude of the effect compared to placebo was trivial (*d* = 0.06–0.33), failing to reach statistical significance in between-group analyses. Furthermore, unspecified *L. plantarum* strains demonstrated no discernible influence on CMJ metrics.

In summary, the influence of *L. plantarum* on muscular strength is highly strain-contingent. PS128 exhibits consistent efficacy in safeguarding against post-exercise strength loss, positioning it as a recovery-oriented strain. The ergogenic response of TWK10 on handgrip strength is jointly modulated by the target population and formulation type (viable vs. postbiotic). PL-02 elicits only marginal between-group benefits, while unspecified strains fail to confer meaningful improvements in muscular performance.

### 3.5 Exercise recovery

#### 3.5.1 Muscle damage biomarkers

The evaluation of exercise-induced muscle damage (EIMD) focused primarily on enzymatic and structural biomarkers, including creatine kinase (CK), lactate dehydrogenase (LDH), and myoglobin. A total of seven randomized controlled trials (RCTs) reported outcomes related to these indices. The protective efficacy of *L. plantarum* against muscle injury displayed pronounced strain specificity (Table 6).

**TABLE 6:** Summary of study characteristics and effects of different *Lactiplantibacillus plantarum* strains on muscle damage markers outcomes.

Strain	Study (year)	Population	Intervention	Outcome	Main result (vs placebo)	Effect size [Cohen’s *d* (95% CI)]	*p*-value
TWK10	Lee et al. (33)	Healthy adults (*n* = 53)	3 × 10^11^ CFU/d, 6 wk	CK	↔ vs. placebo	(NR)	*P* > 0.05
TWK10-HK	Lee et al. (33)	Healthy adults (*n* = 53)	3 × 10^11^ cells/d, 6 wk	CK	↔ vs. placebo	(NR)	*P* > 0.05
TWK10	Huang et al. (31)	Healthy adults (*n* = 54)	3 × 10^10^/9 × 10^10^ CFU/d, 6 wk	CK	↔ vs. placebo	(NR)	*P* > 0.05
TWK10	Huang et al. (30)	Healthy males (*n* = 16)	1 × 10^11^ CFU/d, 6 wk	CK	↔ vs. placebo	(NR)	> 0.05
TWK10-HK	Cheng et al. (29)	Healthy males (*n* = 30)	3 × 10^10^ cells/d, 6 wk	CK	↔ vs. placebo	(NR)	> 0.05
PS128	Fu et al. (37)	Recreational runners (*n* = 8, half-marathon)	3 × 10^10^ CFU/d, 4 wk	CK	↓ vs. placebo (at 3H/24 h/48 h post-HM)	(NR)	*p* < 0.05
				Myoglobin	↓ vs. placebo (at 0 h/3 h post-HM)	(NR)	*p* < 0.05
				LDH	↔ vs. placebo	(NR)	*P* > 0.05
PS128	Huang et al. (31) Study II	Triathletes (*n* = 16)	3 × 10^10^ CFU/d, 3 wk	CK	↓ vs placebo	−21.96 (−29.63, −14.29)	*p* < 0.0001
				LDH	↔ vs. placebo	−1.94 (−3.13,-0.75)	*P* = 0.192
				Myoglobin	↔ vs. placebo	−1.01 (−2.15, −0.05)	*P* = 0.419
PL-02	Lee et al. (47)	Healthy non-athletes (*n* = 88, 20–40 y)	1.5 × 10^10^ CFU/d, 6 wk	CK	↓ vs. placebo (at 24 h post-EIMD)	−0.61 (−1.21, 0.00)^1^	*p* < 0.05
					↓ vs. placebo (at 48 h post-EIMD)	−0.81 (−1.43, −0.20)^1^	*p* < 0.01

↑, significant increase; ↓, significant decrease; ↔, no significant change; HK, heat-killed; CFU, colony-forming units (viable bacteria count); cells, total bacterial cells (including heat-killed forms); wk, week(s); CK, creatine kinase; LDH, lactate dehydrogenase; HM, half-marathon; EIMD, Exercise-Induced Muscle Damage; CI, confidence interval; NR, not reported (insufficient data to calculate effect size and 95% CI).

^1^ Effect sizes marked with

^1^ were estimated from reported *P*-values and sample sizes due to missing descriptive statistics; unmarked effect sizes were calculated from original means and SDs Cohen’s *d*: small (0.2), medium (0.5), large (0.8).

Based on the available literature, PS128 exhibited a high degree of consistency in the current evidence for mitigating post-exercise muscle damage. In competitive triathletes, supplementation with PS128 (3 × 10^10^CFU/day for 3 weeks) was associated with a reduction in CK elevation, yielding a notable effect size (Cohen’s *d* = -21.96; 95% CI: −29.63 to −14.29). Similarly, in recreational runners, the identical regimen over 4 weeks significantly suppressed the surge in both CK and myoglobin levels following a half-marathon. Although all PS128 trials utilized a fixed-dose design—precluding a dose-response characterization—the protective pattern appeared consistent across intervention periods of 3–4 weeks.

PL-02 likewise exhibited significant potential in facilitating muscle recovery. In healthy non-athletes, a 6-week intervention (1.5 × 10^10^CFU/day) significantly blunted CK levels at 24 h (*d* = -0.61) and 48 h (*d* = -0.81) post-EIMD, indicating enhanced recovery kinetics of muscle cell integrity. Given that only a single trial is available for this strain, the dose-duration-response relationship remains to be further elucidated.

In contrast, TWK10 (including both viable and heat-inactivated formulations) appears to have negligible effects on biomarkers of muscle injury. Four trials, employing dosages ranging from 3 × 10^10^ to 3 × 10^11^ CFU/day over 6 weeks, consistently failed to identify significant inter-group differences in CK levels post-intervention (*P* > 0.05). These data suggest that the alleviation of structural muscle damage is likely not a primary biological function of the TWK10 strain.

In summary, the protective effects of *L. plantarum* against exercise-induced muscle injury appear to be highly strain-specific. Based on the available exploratory data, PS128 provides the most consistent trends for structural protection; PL-02 displays clear potential for accelerating muscle recovery; Conversely, current evidence for TWK10 has not yet shown a significant advantage in attenuating biochemical markers of muscle damage.

#### 3.5.2 Inflammatory and oxidative stress markers

A total of six randomized controlled trials (RCTs) reported biomarkers associated with systemic inflammation and oxidative stress, encompassing pro- and anti-inflammatory cytokines (TNF-α, IL-6, IL-8, IL-10), systemic inflammatory indices (Neutrophil-to-Lymphocyte Ratio, NLR; Platelet-to-Lymphocyte Ratio, PLR), and antioxidant enzymes (Superoxide Dismutase, SOD; Catalase, CAT). The findings further substantiate that the immunomodulatory and antioxidant properties of *L. plantarum* are characterized by pronounced strain specificity (Table 7).

**TABLE 7:** Summary of study characteristics and effects of different *Lactiplantibacillus plantarum* strains on inflammatory markers outcomes.

Strain	Study (year)	Population	Intervention	Outcome	Main result	Effect size [Cohen’s *d* (95% CI)]	*p*-value
PS128	Huang et al. (31)	Triathletes (*n* = 16)	3 × 10^10^ CFU/d, 3 wk	TNF-α at AfterEx	↓ vs. placebo	−3.19 (−4.66, −1.72)	*p* < 0.016
				IL-6 at AfterEx	↓ vs. placebo	−3.10 (−4.55, −1.65)	*p* < 0.016
				IL-8 at AfterEx	↓ vs. placebo	−3.08 [(4.53, −1.63)	*p* < 0.016
				IL-10 at AfterEx	↑ vs. placebo	2.92 (1.51, 4.33)	*p* < 0.016
PS128	Fu et al. (37)	Recreational runners (*n* = 8)	3 × 10^10^ CFU/d, 4 wk	SOD (3–96 h post-HM)	↑ vs. placebo	(NR)	*p* < 0.05
				CAT	↔ vs. placebo	(NR)	*P* > 0.05
TWK10	Lee et al. (33)	Healthy adults (*n* = 53, 20–30 y)	3 × 10^11^ CFU/d, 6 wk	NLR/PLR	↔ vs. placebo	(NR)	*p* > 0.05
TWK10-HK	Lee et al. (33)	Healthy adults (*n* = 53, 20–30 y)	3 × 10^11^ cells/d, 6 wk	NLR/PLR	↔ vs. placebo	(NR)	*p* > 0.05
TWK10	Huang et al. (25)	Healthy adults (*n* = 54, 20–30 y)	3 × 10^10^/9 × 10^10^ CFU/d, 6 wk	NLR/PLR	↔ vs. placebo	(NR)	*p* > 0.05
TWK10-HK	Cheng et al. (29)	Healthy males (*n* = 30, 20–40 y)	3 × 10^10^ cells/d, 6 wk	NLR/PLR	↔ vs. placebo	(NR)	*p* > 0.05
PL-02	Lee et al. (47)	Healthy non-athletes (*n* = 88, 20-40 y)	1.5 × 10^10^ CFU/d, 6 wk	IL-6 at 24 h post-EIMD	↓ vs. placebo	(NR)	*p* < 0.05

↑, significant increase; ↓, significant decrease; ↔, no significant change; HK, heat-killed; CFU, colony-forming units (viable bacteria count); cells, total bacterial cells (including heat-killed forms); wk, week(s); NLR, Neutrophil-to-Lymphocyte Ratio; PLR, Platelet-to-Lymphocyte Ratio; SOD, Superoxide dismutase; CAT, Catalase; EIMD, Exercise-Induced Muscle Damage; CI, confidence interval; NR, not reported (insufficient data to calculate effect size and 95% CI).

^1^ Effect sizes marked with

^1^ were estimated from reported *P*-values and sample sizes due to missing descriptive statistics; unmarked effect sizes were calculated from original means and SDs Cohen’s *d*: small (0.2), medium (0.5), large (0.8).

PS128 exhibited a highly consistent trend in modulating the post-exercise inflammatory response. In competitive triathletes, supplementation with PS128 (3 × 10^10^ CFU/day for 3 weeks) significantly suppressed the elevation of pro-inflammatory cytokines, including TNF-α, IL-6, and IL-8, following exhaustive exercise (effect sizes: −3.19 to −3.08), while concurrently augmenting the anti-inflammatory cytokine IL-10(*d* = 2.92). Furthermore, in recreational runners, the identical dosage over 4 weeks significantly enhanced SOD activity, suggesting a role in mitigating exercise-induced oxidative insults. Although the reliance on fixed-dose designs precluded a formal dose-response analysis, the immunomodulatory signature of PS128 appeared consistent across intervention periods of 3–4 weeks.

PL-02 demonstrated discernible anti-inflammatory potential. In healthy non-athletes, PL-02 supplementation (1.5 × 10^10^ CFU/day for 6 weeks) significantly attenuated IL-6 levels at 24 h post-exercise-induced muscle damage (EIMD). This reduction aligns with the observed recovery kinetics of muscle damage markers, suggesting that PL-02 may facilitate recovery by dampening the secondary inflammatory cascade associated with muscle injury.

In contrast, TWK10 (encompassing both viable and postbiotic formulations) exhibited negligible regulatory impact on systemic inflammatory markers. Three trials (dosing range: 3 × 10^10^ to 3 × 10^11^ CFU/day for 6 weeks) consistently failed to identify significant inter-group differences in NLR or PLR (*P* > 0.05). These results indicate that systemic anti-inflammation and antioxidant modulation are likely not primary functional domains of the TWK10 strain.

In summary, the regulation of inflammation and oxidative stress by *L. plantarum* is highly strain-contingent. PS128 exhibits the most consistent trends, characterized by a potential dual regulatory mechanism that simultaneously suppresses pro-inflammatory signaling and bolsters anti-inflammatory and antioxidant defenses. PL-02 confers preliminary, targeted anti-inflammatory protection by modulating IL-6 kinetics, whereas current evidence for TWK10 has not yet demonstrated substantial immunomodulatory efficacy in the context of exercise.

#### 3.5.3 Metabolic and neuromuscular function

The physiological mechanisms through which *L. plantarum* mitigates fatigue encompass both systemic metabolic recovery and the preservation of neuromuscular integrity. The existing evidence suggests a distinct mechanistic divergence between the TWK10 and PS128 strains (Table 8).

**TABLE 8:** Summary of study characteristics and effects of different *Lactiplantibacillus plantarum* strains on recovery markers outcomes.

Strain	Study (year)	Population	Intervention	Outcome	Main result	Effect size [Cohen’s *d* (95% CI)]	*p*-value
TWK10	Huang et al. (30)	Healthy males (*n* = 16, 20–40 y, untrained)	1 × 10^11^ CFU/d, 6 wk	Lactate (post-exercise)	↔ vs. placebo	(NR)	*p* > 0.05
				Ammonia (post-exercise)	↔ vs. placebo	−0.22 (−1.20, 0.76)	*p* > 0.05
				Glucose (post-exercise)	↑ vs. placebo	−3.00 (−4.48, −1.52)	*P* < 0.05
TWK10	Huang et al. (25)	Healthy adults (*n* = 54, 20–30 y, untrained)	3 × 10^10^/9 × 10^10^ CFU/d, 6 wk	Lactate (E30, R20, R40, R60)	↓ vs. placebo	(NR)	*P* < 0.05
				Ammonia (E15, E30, R20)	↓ vs. placebo	(NR)	*P* < 0.05
				Glucose (E15, E30)	↑(high dose) vs. placebo	(NR)	*P* < 0.05
TWK10	Lee et al. (33)	Healthy adults (*n* = 53, 20–30 y, untrained)	3 × 10^11^ CFU/d, 6 wk	Lactate (E10, E15, E30, R20, R40)	↓ vs. placebo	(NR)	*P* < 0.05
				Ammonia (E10, E15, E30, R20, R40)	↓ vs. placebo	(NR)	*P* < 0.05
				Glucose (E15, E30, R20)	↑ vs. placebo	(NR)	*P* < 0.05
TWK10-HK	Lee et al. (33)	Healthy adults (*n* = 53, 20–30 y, untrained)	3 × 10^11^ cells/d, 6 wk	Lactate (E10, E15, E30, R20, R40)	↓ vs. placebo	(NR)	*P* < 0.05
				Ammonia (E15, E30, R20, R40)	↓ vs. placebo	(NR)	*P* < 0.05
				Glucose	↔ vs. placebo	(NR)	*P* > 0.05
TWK10-HK	Cheng et al. (29)	Healthy males (*n* = 30, 20–40 y, untrained)	3 × 10^10^ cells/d, 6 wk	Lactate (E15, E30, R20)	↓ vs. placebo	(NR)	*P* < 0.05
				Ammonia (E30, R60)	↓ vs. placebo	(NR)	*P* < 0.05
PS128	Fu et al. (37)	Recreational runners (*n* = 8, half-marathon)	3 × 10^10^ CFU/d, 4 wk	BCAA	↔ vs. placebo	(NR)	*p* > 0.05
				NH3	↔ vs. placebo	(NR)	*p* > 0.05
PS128	Yu et al. (35)	Recreational runners (*n* = 8, half-marathon)	3 × 10^10^ CFU/d, 4 wk	MDF	↑ vs. placebo (at 0 h post-HM)	1.85 (0.68, 3.02)^1^	*P* < 0.0022
				iEMG	↑ vs. placebo (at 24 h post-HM)	2.07 (0.86, 3.28)^1^	*P* < 0.0001
				NME	↔ vs. placebo	(NR)	*p* > 0.05

↑, significant increase; ↓, significant decrease; ↔, no significant change; HK, heat-killed; CFU, colony-forming units; wk, week(s); E, exercise phase; R, recovery phase; HM, half-marathon; BCAA, branched-chain amino acids; MDF, median frequency; iEMG, integrated electromyography; NME, neuromuscular efficiency; CI, confidence interval; NR, not reported (insufficient data to calculate effect size and 95% CI).

^1^Effect sizes marked with

^1^ were estimated from reported *P*-values and sample sizes due to missing descriptive statistics; unmarked effect sizes were calculated from original means and SDs Cohen’s *d*: small (0.2), medium (0.5), large (0.8).

TWK10 (including its postbiotic form) Effectively enhanced post-exercise metabolic kinetics by accelerating the clearance of lactate and blood ammonia while stabilizing blood glucose levels during exertion. In healthy adults, a 6-week intervention (3 × 10^10^ CFU/day) resulted in significantly lower circulating lactate and ammonia concentrations during both exercise and recovery phases compared to placebo cohorts. This ergogenic effect suggested a dose-dependent trend, with higher dosages yielding more evident metabolic clearance, reinforcing the premise that metabolic optimization is a primary functional domain of TWK10. Notably, the heat-inactivated formulation (postbiotic) effectively retained these metabolic benefits, demonstrating no significant disparity in clearance efficiency relative to the viable strain. A small-scale study by Huang et al. (30) reported only modest elevations in blood glucose without significant changes in lactate or ammonia, which was likely attributable to insufficient statistical power inherent in its limited sample size.

In contrast, PS128 demonstrated negligible influence on systemic metabolic fatigue markers. In recreational runners, a 4-week supplementation regimen (3 × 10^10^ CFU/day) failed to modulate serum ammonia or branched-chain amino acid (BCAA) levels following a half-marathon. However, at the neuromuscular interface, PS128 exhibited distinct protective efficacy. Immediately post-exercise, the decline in median frequency (MDF) of the vastus medialis was significantly attenuated compared to placebo (*d* = 1.85). Furthermore, during the 24–72 h recovery window, integrated electromyography (iEMG) levels remained significantly elevated (*d* = 2.07). These findings suggest that PS128 facilitates recovery by maintaining EMG spectral integrity and alleviating exercise-induced neuromuscular conduction fatigue. While the reliance on fixed-dose designs precluded dose-response characterization, the neuromuscular protective signature of PS128 remained consistent across the 4-week intervention protocols.

In summary, the anti-fatigue mechanisms of these two strains appear to be characteristically distinct: TWK10 acts as a metabolic modulator, significantly enhancing the clearance of fatigue-related metabolites (lactate/ammonia) in a dose-dependent manner; whereas PS128 functions as a neuromuscular stabilizer, prioritizing the maintenance of electromyographic activity and neural drive over systemic metabolic regulation.

### 3.6 Gut microbiota and metabolic profiles

A total of three studies characterized the impact of *L. plantarum* supplementation on gut microbiota composition and short-chain fatty acid (SCFA) profiles. These investigations were primarily small-scale (*n* = 20–53), single-center trials utilizing 16S rRNA gene or metagenomic shotgun sequencing. Collectively, TWK10, PS128, and PL-02 exhibited the potential to restructure the intestinal microbial architecture, selectively enriching beneficial taxa and modulating SCFA profiles through strain-specific regulatory patterns.

Following a 6-week intervention with viable TWK10, an expansion in the relative abundance of *Akkermansiaceae* and *Prevotellaceae* was observed, concurrently with a significant elevation in fecal acetate concentrations (*P* < 0.05) and a notable trend toward increased butyrate (*P* = 0.074). Interestingly, heat-inactivated TWK10 (postbiotic) significantly increased the abundance of the Proteobacteria phylum (*P* = 0.030) and elevated both acetate and propionate levels. Functional metagenomic profiling suggested that viable TWK10 was enriched in glutathione and ubiquinone metabolism, whereas the postbiotic formulation primarily modulated amino acid metabolic pathways.

A 4-week supplementation with PS128 significantly reduced α-diversity indices in competitive triathletes while inducing a distinct shift inβ-diversity structure (*P* < 0.05). At the genus level, PS128 significantly enriched the populations of *Akkermansia*, *Bifidobacterium*, and *Butyricimonas* (all *P* < 0.05), while suppressing potential pro-inflammatory taxa such as *Anaerotruncus* (*P* < 0.05). Correspondingly, fecal concentrations of acetate, propionate, and butyrate were significantly higher in the PS128 cohort compared to placebo (all *P* < 0.05), suggesting a distinct enhancement of the colonic fermentation environment.

Regarding PL-02, a 6-week intervention significantly promoted the expansion of *Lactiplantibacillus* and butyrate-producing *Lachnospiraceae* (all *P* < 0.01), while reducing the abundance of the opportunistic pathogen Sutterella (*P* < 0.01). However, fecal SCFA concentrations were not quantified in this specific trial.

In summary, TWK10, PS128, and PL-02 share a convergent regulatory axis characterized by the enrichment of SCFA-producing commensals, notably *Akkermansia*, *Bifidobacterium*, and the *Lachnospiraceae* family. Nevertheless, the current evidence is derived exclusively from descriptive associative analyses, which precludes the establishment of a causal nexus between gut microbiota modulation and the observed ergogenic phenotypes.

### 3.7 Safety and tolerability

All 12 included randomized controlled trials (RCTs) provided systematic safety monitoring data and reported no serious adverse events (SAEs) associated with *L. plantarum* supplementation. Specifically, blood biochemical profiles remained within physiological ranges, indicating that the interventions did not perturb systemic metabolic or organ-function homeostasis. Furthermore, gastrointestinal tolerance was characterized as excellent, with no reports of persistent bloating, abdominal distress, or altered bowel habits across the diverse cohorts.

Collectively, these findings demonstrate that *L. plantarum* supplementation, within a dosage range of 1 × 10^9^ to 3 × 10^11^ CFU/day for short-to-medium durations (3–18 weeks), is safe and well-tolerated in healthy populations, including athletes and elderly individuals.

## 4 Discussion

### 4.1 Summary of key findings

First, the biological efficacy of *L. plantarum* in exercise science is characterized by distinct strain-specificity, with distinct strains exhibiting divergent functional roles and ergogenic profiles.

. Current literature suggests that TWK10 may improve aerobic endurance and metabolic recovery, though this is based on a limited cluster of trials. Four out of five trials confirmed that TWK10 significantly bolsters aerobic capacity, with Time to Exhaustion (TTE) increasing by 1.24–1.58-fold compared to placebo. This ergogenic effect follows a clear dose-dependent trajectory, with high-dose regimens (9 × 10^10^ CFU/day) yielding superior performance gains. Regarding recovery, current data suggest that TWK10 accelerates the clearance of blood lactate and ammonia, achieving homeostatic stability even at lower dosages (3 × 10^10^ CFU/day). Notably, the heat-inactivated formulation (postbiotic) retains metabolic recovery benefits comparable to its viable counterpart. Interestingly, TWK10 lacks significant impact on structural muscle damage (CK) or systemic inflammatory indices (NLR/PLR), suggesting a high degree of metabolic pathway selectivity. However, TWK10’s efficacy is likely constrained by host metabolic status and sex, given its lack of ergogenic impact in obese middle-aged females. Obesity-associated low-grade inflammation or insulin resistance may blunt strain-mediated signaling, while age- and sex-specific endocrine profiles (e.g., estrogen dynamics) could differentially regulate skeletal muscle metabolism. These discrepancies underscore host intrinsic factors as critical modifiers of L. plantarum efficacy.

PS128 displays a unique functional signature centered on immunomodulation and neuromuscular preservation. Its efficacy in enhancing aerobic and anaerobic performance appears to be performance-contingent, conferring significant benefits primarily in elite athletes while remaining largely ineffective in recreational cohorts. Nonetheless, PS128 shows a notable trend toward facilitating recovery, with preliminary data indicating it can mitigate EIMD (as evidenced by substantial CK reduction), preserves neuromuscular integrity (MVIC, CMJ), and orchestrates the cytokine cascade by suppressing pro-inflammatory signaling (TNF-α, IL-6, IL-8) while upregulating the anti-inflammatory mediator IL-10. Although limited by fixed-dose designs (3 × 10^10^ CFU/day), PS128 elicits stable protective effects within a relatively short intervention window of 3–4 weeks.

PL-02, as an emerging candidate, has demonstrated multidimensional potential for exercise recovery. Evidence from high-quality RCTs indicates that PL-02 (1.5 × 10^10^ CFU/day) attenuates biochemical markers of muscle injury (CK, d = -0.64 to −0.82), and modulates IL-6 kinetics. However, the inter-group effects of PL-02 on aerobic, anaerobic capacity and muscular strength-related parameters remained marginal, failing to reach statistical significance. Given the current reliance on a single primary study, these findings warrant further independent validation.

Notably, *L. plantarum* preparations with unspecified strains tended not to yield demonstrable ergogenic or physiological benefits, further reinforcing that strain-specificity is the primary determinant of intervention efficacy.

Second, dose-response relationships and optimal intervention timelines appear to be both outcome-dependent and strain-specific. TWK10 is the only strain supported by dose-gradient data, revealing that while endurance enhancement is dose-dependent, metabolic clearance efficiency may plateau at lower dosages. PS128 lacks dose-response characterization due to uniform dosing protocols across the literature, yet it demonstrates a consistent temporal efficacy within a 4-week framework. For PL-02 and unspecified formulations, the scarcity of data precludes the identification of optimal therapeutic windows.

Third, the modulation of the gut microbiota serves as a convergent core mechanism through which these distinct strains improve exercise phenotypes. TWK10, PS128, and PL-02 collectively restructure the microbial landscape, notably enriching short-chain fatty acid (SCFA)-producing taxa—including *Akkermansia*, Bifidobacterium, and the *Lachnospiraceae* family—while concurrently elevating fecal levels of acetate, propionate, and butyrate. Despite strain-specific variations in their primary molecular targets, the maintenance of intestinal microecological homeostasis emerges as the common regulatory axis mediating enhanced physical performance and accelerated fatigue recovery.

### 4.2 Potential biological mechanisms

High-intensity or prolonged physical exertion frequently precipitates systemic homeostatic perturbations, characterized by skeletal muscle metabolic imbalances, the accumulation of deleterious by-products (e.g., lactate, ammonia), intestinal barrier dysfunction (“leaky gut”), and exacerbated systemic inflammatory cascades. These factors collectively impair acute physical performance and impede long-term recovery, with exercise-induced gut dysbiosis serving as a pivotal driver of these pathophysiological processes (51–53). This pathophysiological cascade highlights the relevance of microbiota-targeted strategies; for instance, recent randomized data demonstrate that synbiotic supplementation can mitigate post-exercise surges in circulatory cytokines and muscle-damage biomarkers in athletic cohorts (54).

The ameliorative effects of distinct *L. plantarum* strains on exercise performance and recovery kinetics exhibit significant functional heterogeneity, primarily stemming from their divergent regulatory signatures along the “gut-muscle axis” and “gut-immune axis.” Specifically, TWK10 appears to prioritize the gut-muscle axis by optimizing skeletal muscle substrate utilization and accelerating the clearance of metabolic waste, thereby enhancing endurance capacity. Conversely, PS128 primarily targets the gut-immune axis to mitigate systemic inflammation and preserve neuromuscular integrity during recovery. Together, these strains synergistically expand the nutritional regulatory repertoire of L. plantarum in exercise science (55–58).

**FIGURE 5:**
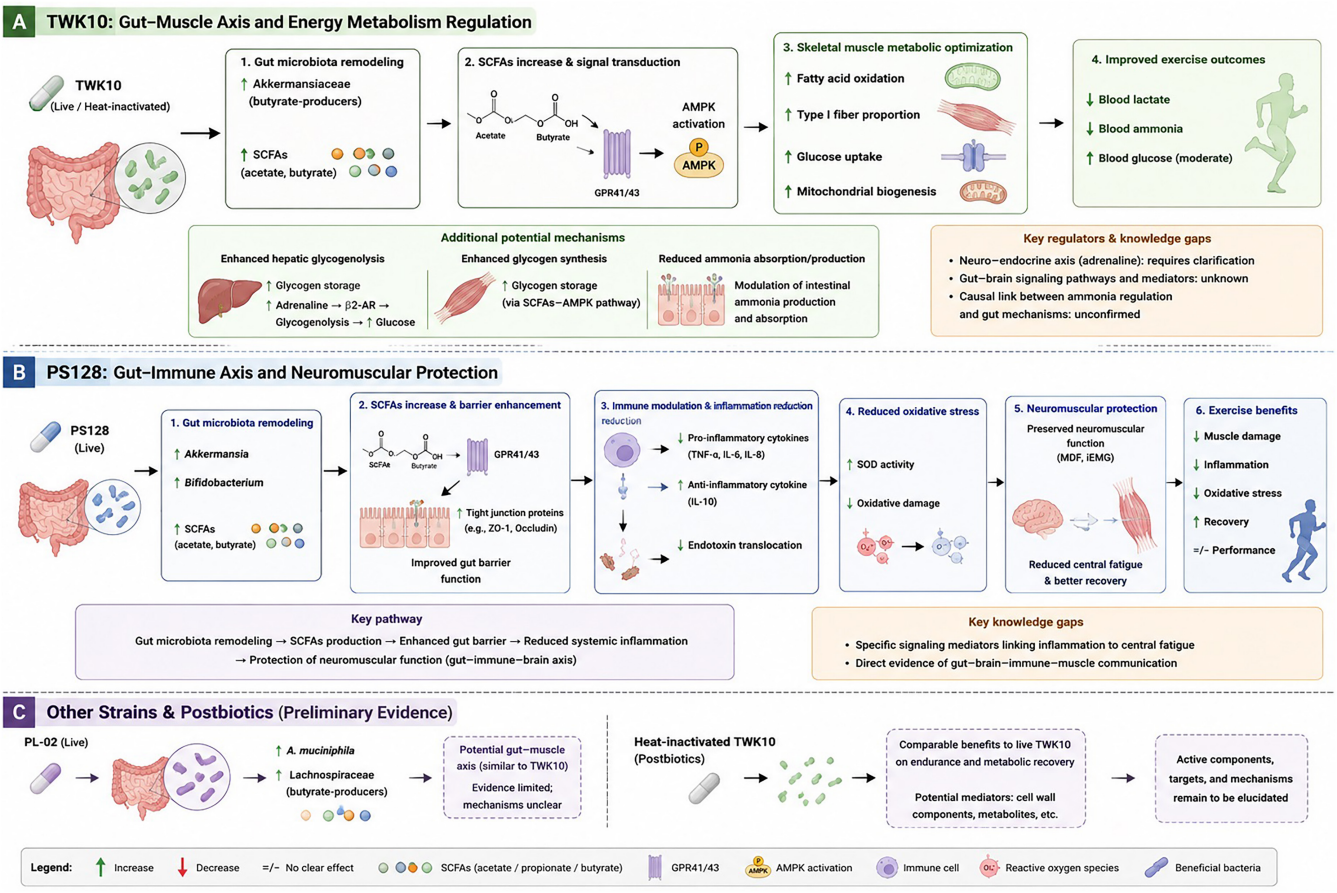
Mechanism of different *Lactiplantibacillus plantarum* strains. **(A)** TWK10. **(B)** PS128. **(C)** Other strains & postbiotics.

Notably, evidence suggests that these regulatory pathways may intersect at a common molecular foundation: the remodeling of the intestinal microbial architecture and the subsequent enrichment of short-chain fatty acids (SCFAs). These two axes are highly interconnected, as SCFAs function as dual-functional mediators: they serve both as energetic substrates for skeletal muscle mitochondrial metabolism and as active signaling molecules that modulate inflammatory responses via G protein-coupled receptor (GPCR) signaling in immune cells (59–61). This specific “strain-pathway-phenotype” association not only elucidates the core mechanistic landscape through which *L. plantarum* exerts its ergogenic effects but also establishes a robust theoretical foundation for the implementation of precision nutrition frameworks in athletic populations (Figure 5).

#### 4.2.1 TWK10: the gut–muscle axis and regulation of energy metabolism

Following TWK10 supplementation, the most consistent physiological signatures observed include markedly attenuated blood lactate and ammonia concentrations, alongside a moderate elevation in circulating glucose during exercise (29, 30, 33). This metabolic profile suggests that the core ergogenic mechanism of TWK10 resides in the optimization of substrate utilization efficiency within skeletal muscle and the accelerated clearance of exercise-induced metabolic by-products.

The reduction in lactate levels is hypothesized to stem from a metabolic shift favoring fatty acid oxidation over anaerobic glycolysis. Preclinical evidence has confirmed that TWK10 upregulates key enzymatic proteins involved in hepatic lipid oxidation and induces a phenotypic shift toward Type I (slow-twitch) muscle fibers (23, 24). Given that the aerobic metabolic machinery of Type I fibers inherently facilitates higher lactate clearance rates (62), this structural adaptation provides a clear biological basis for the observed endurance gains. Consequently, TWK10 likely delays the onset of muscular fatigue through a dual-action pathway: enhancing lipid oxidative capacity and promoting myofiber transformation to suppress lactate accumulation.

The exercise-induced glycemic response observed with TWK10 is presumed to be linked to augmented hepatic glycogenolysis, representing another pivotal ergogenic facet of this strain. In murine models, TWK10 significantly bolstered hepatic and skeletal muscle glycogen reserves in a dose-dependent manner (23, 63). This glycogen modulation is likely mediated via the gut–muscle axis rather than direct action; specifically, the restructuring of the gut microbiota and subsequent elevation of short-chain fatty acids (SCFAs) activate downstream signaling pathways that favor glycogen synthesis and storage (34). Furthermore, clinical data suggest that the glycemic surge may be partially driven by the upregulation of epinephrine secretion (33). The classical neuro-endocrine mechanism, wherein catecholamines stimulate hepatic glucose output via β_2_–adrenoceptor activation, is well-established (64, 65). Notably, the glycemic response was significantly more pronounced in the viable TWK10 group compared to its heat-inactivated counterpart. This suggests that certain regulatory effects—particularly those involving the gut–brain–endocrine axis—may be contingent upon live bacteria-mediated signaling, representing a critical frontier for future mechanistic inquiry.

Regarding nitrogenous waste, both low- and high-dose TWK10 interventions effectively lowered circulating ammonia with no discernible dose-response disparity (31). This indicates that ammonia mitigation is a fundamental functional trait of the strain. It is hypothesized that TWK10 may attenuate systemic ammonia levels by modulating its intestinal biogenesis or absorption (33), although targeted tracer experiments are required to confirm this causal link.

At the molecular level, the synergistic regulation of SCFAs and the AMPK pathway constitutes the cornerstone of the TWK10-mediated gut–muscle axis. Supplementation significantly enriches butyrate-producing *Akkermansiaceae* and elevates fecal acetate and butyrate levels (33). Butyrate serves as both a secondary energy substrate and a potent signaling molecule (66). Crucially, SCFAs activate the AMPK (AMP-activated protein kinase) signaling pathway, the “metabolic master switch” in skeletal muscle (59). AMPK activation promotes glucose uptake, stimulates mitochondrial biogenesis, and enhances fatty acid oxidation (67), effectively bridging the gap between intestinal microbial activity and physical performance.

In summary, the TWK10 strain shows potential in supporting energy supply efficiency and mitigating metabolic distress through a structured gut–muscle axis regulatory cascade: Gut microbiota remodeling →Augmented SCFA production → Activation of the AMPK signaling pathway → Optimization of skeletal muscle energy metabolism and fiber-type adaptation.

#### 4.2.2 PS128: the gut–immune axis and neuromuscular protection

In stark contrast to the metabolic focus of TWK10, the biological efficacy of the PS128 strain is predominantly characterized by its potent anti-inflammatory, antioxidant, and neuromuscular preservative properties. These two strains exhibit a sophisticated functional complementarity, collectively diversifying the nutritional regulatory landscape of *L. plantarum* in sports science.

The orchestration of systemic inflammatory responses is a hallmark of PS128. Following supplementation, circulating levels of pro-inflammatory cytokines—notably TNF-α, IL-6, and IL-8—are significantly attenuated, while the primary anti-inflammatory mediator IL-10 is markedly upregulated (68). The anti-inflammatory action of PS128 likely results from the synergistic interplay between intestinal barrier reinforcement and direct immunomodulation. Strenuous exercise frequently precipitates intestinal barrier dysfunction (“leaky gut”) and subsequent endotoxin (LPS) translocation, which triggers systemic inflammatory cascades (9, 69). PS128 bolsters the mucosal barrier to mitigate this endotoxin leakage. Furthermore, in vitro evidence confirms that PS128 can directly inhibit pro-inflammatory signaling pathways (70), a finding corroborated by clinical trials in recreational cohorts (37).

Regarding oxidative stress modulation, PS128 supplementation significantly fortifies the antioxidant defense system, specifically enhancing superoxide dismutase (SOD) activity (37). This antioxidant capacity acts synergistically with its anti-inflammatory effects to neutralize reactive oxygen species (ROS) induced by high-intensity training (71), thereby shielding skeletal muscle and systemic tissues from oxidative damage and accelerating the recovery kinetics of physiological homeostasis.

A unique advantage of PS128 lies in its safeguarding of neuromuscular integrity. Research has demonstrated that PS128 effectively prevents the post-exercise decline in Median Frequency (MDF) and Integrated Electromyography (iEMG) of the vastus medialis following a half-marathon (35). Central nervous system (CNS) fatigue appears to play a substantial role in driving fatigue during endurance disciplines, characterized by impaired neural drive and reduced spinal motor neuron firing rates (72, 73). It is hypothesized that PS128 alleviates CNS-mediated fatigue by attenuating the neuro-inhibitory impact of circulating pro-inflammatory cytokines on the neuromuscular junction and neural transmission pathways, thereby maintaining electromyographic spectral stability during exhaustive exertion.

The modulation of short-chain fatty acids (SCFAs) and the preservation of intestinal integrity constitute the pivotal link in the PS128-mediated gut–immune–brain axis. Intervention with PS128 significantly enriches *Akkermansia* and Bifidobacterium, concurrently elevating fecal SCFA levels (32). *Akkermansia* is renowned for enhancing intestinal barrier function by upregulating tight junction proteins (74), while *Bifidobacterium* synergistically boosts SCFA production. These SCFAs regulate immune cell differentiation and suppress cytokine secretion by activating G protein-coupled receptors (GPR41, GPR43) (60), effectively translating microbial signals into systemic anti-inflammatory protection.

In summary, PS128 exerts its ergogenic and recovery-promoting effects through a multi-level regulatory cascade along the gut–immune–neuromuscular axis: Gut microbiota remodeling → Augmented SCFA production → Enhanced intestinal barrier integrity → Attenuation of systemic inflammation and oxidative stress → Preservation of neuromuscular function and neural drive. This pathway distinguishes PS128 as a specialized “recovery-oriented” strain, linking immunological modulation directly to functional neural stability.

#### 4.2.3 Emerging evidence of novel strains and postbiotics

Beyond the established ergogenic profiles of TWK10 and PS128, evidence regarding the potential of *L. plantarum* PL-02 remains in a nascent stage of exploration. Preliminary data indicate that PL-02 supplementation significantly expands the intestinal populations of *Akkermansia muciniphila* (49) and enriches butyrate-producing *Lachnospiraceae* (47). These findings suggest that PL-02 may draw mechanistic parallels with TWK10, potentially modulating skeletal muscle energy metabolism via a similar gut–muscle axis. However, its precise molecular targets, dose-response dynamics, and broader clinical applicability necessitate further validation. While its capacity to augment butyrate-producing taxa is documented, its direct association with the AMPK signaling cascade remains speculative. Currently, the relative paucity of comparative clinical trials precludes a definitive functional differentiation between PL-02 and TWK10.

The investigation into postbiotics represents a transformative frontier in sports nutrition. Research has demonstrated that heat-inactivated TWK10 exhibits interventional efficacy comparable to its viable counterpart in potentiating endurance and accelerating metabolic recovery (29, 33). This aligns with emerging consensus that inactivated microbial cells, cell fractions, and their metabolites possess potent functional properties capable of modulating host physiological homeostasis (75). The underlying mechanism likely pertains to the unique pharmacodynamic advantages of postbiotic preparations: compared to live bacteria, inactivated microbial components may more efficiently penetrate the intestinal mucus layer and provide stable, persistent stimulation to diverse mucosal cell types (76–78).

These observations imply that the ergogenic benefits of TWK10 may be partially mediated by its structural components—such as cell wall peptidoglycans or lipoteichoic acids—acting as microbial-associated molecular patterns (MAMPs), or by stable metabolites, rather than necessitating active gastrointestinal colonization. While these findings establish a preliminary theoretical framework for postbiotic application in athletic cohorts, the specific bioactive ligands, molecular receptors, and signaling pathways through which heat-inactivated TWK10 elicits its effects remain to be fully elucidated.

### 4.3 Limitations and future perspectives

The present systematic review is subject to several methodological and evidence-related constraints that warrant cautious interpretation of the findings.

First, there is a pronounced lack of geographical and demographic diversity within the current evidence base. Specifically, 11 of the 12 included studies were conducted in Taiwan, China, with participants predominantly consisting of healthy young adults or recreational exercisers. Data concerning elite athletic cohorts and diverse ethnic or geographical populations remain scarce, thereby restricting the generalizability of the synthesized conclusions to broader global populations. Moreover, poorly controlled baselines—including habitual diet, training volume, and initial microbiota composition—may drive differential colonization resistance and metabolic responsiveness to *L. plantarum*, forming another caveat to generalizability.

Second, substantial methodological and clinimetric heterogeneity exists across the identified literature. Discrepancies in exhaustive exercise protocols, muscle strength assessment modalities, and bioinformatic workflows for microbiota sequencing precluded the conduct of a quantitative meta-analysis. This variability mandated a narrative synthesis, which inherently limits the ability to quantify precise pooled effect sizes.

Third, the evidentiary hierarchy for causal inference remains limited. Although the majority of studies identified significant correlations between intestinal microbial architecture and exercise phenotypes, these findings are largely derived from descriptive, associative analyses. There is a notable absence of causal validation utilizing longitudinal interventions, fecal microbiota transplantation (FMT) models, or integrated multi-omics frameworks to mechanistically elucidate the bidirectional signaling within the gut-muscle axis.

Fourth, longitudinal data and granular subgroup analyses are markedly lacking. Only one trial featured an intervention period exceeding 12 weeks, and the absence of stratified analyses based on sex, biological age, or training status obscures individual variability. Crucially, the inclusion of community-dwelling older adults with Clinical Frailty Scale (CFS) scores of 1–4 introduces subtle baseline heterogeneity alongside younger athletic cohorts. In younger populations, *L. plantarum* primarily optimizes metabolic output and fatigue kinetics, whereas in early-aging cohorts, its ergogenic value likely shifts toward suppressing low-grade inflammation and preserving functional mobility via the gut-muscle axis. Failing to stratify these distinct physiological baselines prevents the isolation of age-specific pooled effect sizes. Meanwhile, the null findings reported by Chen et al. (36) in a cohort of obese females suggest that biological sex and adiposity status may act as critical effect modifiers, potentially modulating the ergogenic responsiveness to *L. plantarum*.

Fifth, a formal evidence evaluation using the GRADE framework was not undertaken. Given the pronounced clinical heterogeneity and small sample sizes across primary trials—which would inherently downgrade the evidence across multiple domains—a detailed descriptive appraisal was prioritized over rigid quantitative grading.

Finally, the exclusion of grey literature may introduce a risk of publication bias. However, given the consistent reporting of favorable safety profiles across the included RCTs, the absence of unpublished negative results is unlikely to fundamentally alter the overall direction or reliability of the present findings.

### 4.4 Recommendations for future research

First, strain-specific dose-response dynamics and temporal efficacy must be systematically established. While the dose-dependent efficacy of TWK10 is well-documented, the clinical data for PS128 and PL-02 rely exclusively on single fixed-dose protocols, which precludes the identification of optimal therapeutic windows. Future investigations should implement multi-arm dose-ranging designs and extend intervention durations beyond 12 weeks, incorporating post-intervention follow-up periods to characterize the persistence of ergogenic effects and long-term safety profiles.

Second, there is an urgent need to diversify study populations and standardize assessment frameworks. Current evidence is disproportionately concentrated on healthy young adults and recreational exercisers within restricted geographical regions, with a profound underrepresentation of female cohorts and a complete absence of data on elite athletic populations. Future research should prioritize ethnogeographical diversity and conduct robust subgroup analyses stratified by sex, biological age, and baseline training status. Furthermore, we recommend the development of a Core Outcome Set (COS) for probiotic interventions in sports nutrition—encompassing aerobic endurance, muscle damage kinetics, inflammatory cytokine profiles, and fecal/serum microbial metabolites—to minimize heterogeneity and strengthen the rigor of future meta-analytical syntheses.

Third, research must transition from descriptive associations toward mechanistic causality. As current microbiome analyses remain predominantly associative, future RCTs should integrate multi-omics approaches (e.g., metagenomics and metabolomics) or utilize fecal microbiota transplantation (FMT) and germ-free (GF) animal models to delineate the “microbiota-metabolite-phenotype” causal chain. Additionally, head-to-head trials between viable and heat-inactivated TWK10 are warranted to further quantify the independent molecular efficacy and cost-effectiveness of postbiotic-based interventions.

## 5 Conclusion

In conclusion, supplementation with *L. plantarum* serves as an effective strategy to enhance exercise performance and facilitate post-exercise recovery in healthy populations, although these effects are fundamentally strain-dependent.

- TWK10 exhibits high evidentiary consistency as a specialized “metabolic recovery and endurance” strain, reliably augmenting aerobic capacity and accelerating the clearance of lactate and ammonia; notably, its heat-inactivated postbiotic form largely preserves these core ergogenic benefits.
- PS128 is characterized as an “immunomodulatory and neuromuscular recovery” strain, capable of mitigating structural muscle damage, suppressing systemic inflammation, and preserving neuromuscular function, although its impact on peak performance remains inconsistent.
- PL-02 demonstrates preliminary potential in attenuating markers of muscle damage and inflammation, though within a limited evidence base.
- In contrast, unspecified strains offer negligible physiological advantages, underscoring that strain specificity is the primary determinant of intervention efficacy.

According to the Cochrane RoB 2 assessment, the overall quality of the available evidence is moderate. Methodological limitations primarily involve insufficient reporting of allocation concealment and a low rate of prospective trial registration. Furthermore, the generalizability of these findings is currently constrained by geographical homogeneity and small-to-medium sample sizes. Future large-scale, multicenter RCTs with extended follow-up are essential to validate these conclusions across globally diverse populations and various athletic disciplines.

## Data Availability

The original contributions presented in the study are included in the article/Supplementary material, further inquiries can be directed to the corresponding author.
