# Supplementary material for: Effects of Lactiplantibacillus plantarum supplementation on exercise performance and recovery: a systematic review and narrative synthesis
Source: Front Nutr. 2026 Jul 15;13:1881322. doi: 10.3389/fnut.2026.1881322 (PMC13415575; doi:10.3389/fnut.2026.1881322)
Supplement: Supplementary file 1 [file Table_1.docx]

**Effects of Lactobacillus plantarum Supplementation on Exercise Performance:A Protocol for a Systematic Review and Narrative Synthesis**

**Search restrictions**: Only studies published in English or Chinese were included. No date restrictions were applied.

**Appendix Table 1 Summary of the PICOS criteria used to identify studies to be included**

| **Parameter** | **Description** |
| --- | --- |
| Population | Healthy adults (≥ 18 years), including sedentary individuals, recreational exercisers, and athletes. Studies involving clinical patient populations or hospitalized individuals were excluded. |
| Intervention | Oral supplementation with any strain of Lactobacillus plantarum (including viable and heat-killed forms) in any dose or duration, as a single ingredient or within a defined multi-strain product where the effect of L. plantarum could be isolated. Interventions where L. plantarum could not be isolated (e.g., vague "probiotic blends") or were non-oral were excluded. |
| Comparator | A matched placebo with no active ingredients known to affect exercise performance or related outcomes. |
| Outcomes | At least one objective measure related to:  • Exercise performance (e.g., VO₂max, time to exhaustion, anaerobic capacity, muscle strength, power);  • Post-exercise recovery (e.g., blood lactate, creatine kinase, inflammatory markers, perceived fatigue);  • Body composition as a secondary outcome in exercise studies (e.g., muscle mass, body fat percentage).  Studies focusing solely on disease endpoints or body composition without an exercise component were excluded. |
| Design | Randomized controlled trials (RCTs), including both parallel and crossover designs. Studies reporting only acute (single-dose) effects were excluded. |

**Appendix Table 2 Search strategy (Date 2026-3-10/11)**

| **PubMed database(** Date 2026-3-10**)** | | |
| --- | --- | --- |
| Search number | Search Details | Results |
| 1 | "Lactobacillus plantarum"[Mesh] OR "Lactobacillus plantarum"[tiab] OR "Lacticaseibacillus plantarum"[tiab] OR "Lactiplantibacillus plantarum"[tiab] OR "TWK10"[tiab] OR "PS128"[tiab] OR "PL-02"[tiab] OR "299v"[tiab] | 10,474 |
| 2 | "Exercise"[Mesh] OR "Sports"[Mesh] OR "Physical Endurance"[Mesh] OR "Muscle Strength"[Mesh] OR "Athletic Performance"[Mesh] OR "Physical Fitness"[Mesh] OR "exercise"[tiab] OR "sports"[tiab] OR "physical activity"[tiab] OR "endurance"[tiab] OR "strength"[tiab] OR "performance"[tiab] OR "recovery"[tiab] OR "fatigue"[tiab] OR "muscle damage"[tiab] OR "creatine kinase"[tiab] OR "lactate"[tiab] OR "VO2max"[tiab] OR "time to exhaustion"[tiab] OR "body composition"[tiab] OR "muscle mass"[tiab] OR "oxidative stress"[tiab] OR "cytokines"[tiab] OR "TNF-α"[tiab] OR "IL-6"[tiab] OR "IL-8"[tiab] OR "IL-10"[tiab] OR "blood ammonia"[tiab] OR "ammonia"[tiab] OR "free fatty acid"[tiab] OR "FFA"[tiab] | 4,432,335 |
| 3 | "Healthy Volunteers"[Mesh] OR "healthy"[tiab] OR "adults"[tiab] OR "adult"[tiab] OR "subjects"[tiab] OR "men"[tiab] OR "women"[tiab] OR "athlete*"[tiab] OR "trained"[tiab] OR "sedentary"[tiab] OR "recreational"[tiab] OR "non-athlete"[tiab] OR "physically active"[tiab] OR "older adults"[tiab] OR "elderly"[tiab] | 5,427,980 |
| 4 | "Randomized Controlled Trial"[pt] OR "Controlled Clinical Trial"[pt] OR "randomized"[tiab] OR "randomised"[tiab] OR "randomly"[tiab] OR "placebo"[tiab] OR "trial"[tiab] OR "RCT"[tiab] | 2,005,138 |
| 5 | #1 AND #2 AND #3 AND #4 | 104 |
| **Web of Science（Date 2026-3-10）** | | |
| 1 | "Lactobacillus plantarum" OR "Lacticaseibacillus plantarum" OR "Lactiplantibacillus plantarum" OR "TWK10" OR "PS128" OR "PL-02" OR "299v" (Topic) | 19,803 |
| 2 | "Exercise" OR "Sports" OR "Physical Endurance" OR "Muscle Strength" OR "Athletic Performance" OR "Physical Fitness" OR "exercise" OR "sports" OR "physical activity" OR "endurance" OR "strength" OR "performance" OR "recovery" OR "fatigue" OR "muscle damage" OR "creatine kinase" OR "lactate" OR "VO2max" OR "time to exhaustion" OR "body composition" OR "muscle mass" OR "oxidative stress" OR "cytokines" OR "TNF-α" OR "IL-6" OR "IL-8" OR "IL-10" OR "blood ammonia" OR "ammonia" OR "free fatty acid" OR "FFA" (Topic) | 12,125,396 |
| 3 | "Healthy Volunteers" OR "healthy" OR "adults" OR "adult" OR "subjects" OR "men" OR "women" OR "athlete*" OR "trained" OR "sedentary" OR "recreational" OR "non-athlete" OR "physically active" OR "older adults" OR "elderly" (Topic) | 6,936,523 |
| 4 | "Randomized Controlled Trial" OR "Controlled Clinical Trial" OR "randomized" OR "randomised" OR "randomly" OR "placebo" OR "trial" OR "RCT" (Topic) | 2,686,357 |
| 5 | #1 AND #2 AND #3 AND #4 | 155 |
| **SCOPUS****（Date 2026-3-10）** | | |
| 1 | TITLE-ABS-KEY ( "lactobacillus plantarum" OR "lacticaseibacillus plantarum" OR "lactiplantibacillus plantarum" OR "twk10" OR "ps128" OR "pl-02" OR "299v" | 24,183 |
| 2 | TITLE-ABS-KEY ( "exercise" OR "sports" OR "physical endurance" OR "muscle strength" OR "athletic performance" OR "physical fitness" OR "exercise" OR "sports" OR "physical activity" OR "endurance" OR "strength" OR "performance" OR "recovery" OR "fatigue" OR "muscle damage" OR "creatine kinase" OR "lactate" OR "vo2max" OR "time to exhaustion" OR "body composition" OR "muscle mass" OR "oxidative stress" OR "cytokines" OR "tnf-α" OR "il-6" OR "il-8" OR "il-10" OR "blood ammonia" OR "ammonia" OR "free fatty acid" OR "ffa" ) | 16,665,149 |
| 3 | TITLE-ABS-KEY ( "healthy volunteers" OR "healthy" OR "adults" OR "adult" OR "subjects" OR "men" OR "women" OR "athlete*" OR "trained" OR "sedentary" OR "recreational" OR "non-athlete" OR "physically active" OR "older adults" OR "elderly" ) | 16,272,836 |
| 4 | TITLE-ABS-KEY ( "Randomized Controlled Trial" OR "Controlled Clinical Trial" OR "randomized" OR "randomised" OR "randomly" OR "placebo" OR "trial" OR "RCT" ) | 4,282,044 |
| 5 | #1 AND #2 AND #3 AND #4 | 365 |
| COCHRANE**（Date 2026-3-11）** | | |
| 1 | MeSH descriptor: [Lactobacillus plantarum] explode all trees | 177 |
| 2 | ("Lactobacillus plantarum" OR "Lacticaseibacillus plantarum" OR "Lactiplantibacillus plantarum" OR "TWK10" OR "PS128" OR "PL-02" OR "299v"):ti,ab,kw | 902 |
| 3 | #1 OR #2 | 902 |
| 4 | MeSH descriptor: [exercise] explode all trees | 42085 |
| 5 | MeSH descriptor: [Sports] explode all trees | 23487 |
| 6 | MeSH descriptor: [Physical Endurance] explode all trees | 7871 |
| 7 | MeSH descriptor: [Muscle Strength] explode all trees | 9981 |
| 8 | MeSH descriptor: [Athletic Performance] explode all trees | 10802 |
| 9 | MeSH descriptor: [Physical Fitness] explode all trees | 5232 |
| 10 | ("exercise" OR "sports" OR "physical endurance" OR "muscle strength" OR "athletic performance" OR "physical fitness" OR "physical activity" OR "endurance" OR "strength" OR "performance" OR "recovery" OR "fatigue" OR "muscle damage" OR "creatine kinase" OR "lactate" OR "VO2max" OR "time to exhaustion" OR "body composition" OR "muscle mass" OR "oxidative stress" OR "cytokines" OR "TNF-α" OR "IL-6" OR "IL-8" OR "IL-10" OR "blood ammonia" OR "ammonia" OR "free fatty acid" OR "FFA"):ti,ab,kw | 490998 |
| 11 | #4 OR #5 OR #6 OR #7 OR #8 OR #9 OR #10 | 493622 |
| 12 | MeSH descriptor: [Healthy Volunteers] explode all trees | 7163 |
| 13 | ("healthy" OR "adults" OR "adult" OR "subjects" OR "men" OR "women" OR athlete* OR "trained" OR "sedentary" OR "recreational" OR "non-athlete" OR "physically active" OR "older adults" OR "elderly"):ti,ab,kw | 1281690 |
| 14 | #12 OR #13 | 1281690 |
| 15 | #3 AND #11 AND #14 | 197 |
| SPORTDiscus（Date 2026-3-11） | | |
| 1 | TI ("Lactobacillus plantarum" OR "Lacticaseibacillus plantarum" OR "Lactiplantibacillus plantarum" OR "TWK10" OR "PS128" OR "PL-02" OR "299v") OR AB ("Lactobacillus plantarum" OR "Lacticaseibacillus plantarum" OR "Lactiplantibacillus plantarum" OR "TWK10" OR "PS128" OR "PL-02" OR "299v") OR KW ("Lactobacillus plantarum" OR "Lacticaseibacillus plantarum" OR "Lactiplantibacillus plantarum" OR "TWK10" OR "PS128" OR "PL-02" OR "299v") | 55 |
| 2 | TI ("exercise" OR "sports" OR "physical endurance" OR "muscle strength" OR "athletic performance" OR "physical fitness" OR "physical activity" OR "endurance" OR "strength" OR "performance" OR "recovery" OR "fatigue" OR "muscle damage" OR "creatine kinase" OR "lactate" OR "VO2max" OR "time to exhaustion" OR "body composition" OR "muscle mass" OR "oxidative stress" OR "cytokines" OR "TNF-α" OR "IL-6" OR "IL-8" OR "IL-10" OR "blood ammonia" OR "ammonia" OR "free fatty acid" OR "FFA") OR AB ("exercise" OR "sports" OR "physical endurance" OR "muscle strength" OR "athletic performance" OR "physical fitness" OR "physical activity" OR "endurance" OR "strength" OR "performance" OR "recovery" OR "fatigue" OR "muscle damage" OR "creatine kinase" OR "lactate" OR "VO2max" OR "time to exhaustion" OR "body composition" OR "muscle mass" OR "oxidative stress" OR "cytokines" OR "TNF-α" OR "IL-6" OR "IL-8" OR "IL-10" OR "blood ammonia" OR "ammonia" OR "free fatty acid" OR "FFA") OR KW ("exercise" OR "sports" OR "physical endurance" OR "muscle strength" OR "athletic performance" OR "physical fitness" OR "physical activity" OR "endurance" OR "strength" OR "performance" OR "recovery" OR "fatigue" OR "muscle damage" OR "creatine kinase" OR "lactate" OR "VO2max" OR "time to exhaustion" OR "body composition" OR "muscle mass" OR "oxidative stress" OR "cytokines" OR "TNF-α" OR "IL-6" OR "IL-8" OR "IL-10" OR "blood ammonia" OR "ammonia" OR "free fatty acid" OR "FFA") | 280,966 |
| 3 | TI ("healthy" OR "adults" OR "adult" OR "subjects" OR "men" OR "women" OR athlete* OR "trained" OR "sedentary" OR "recreational" OR "non-athlete" OR "physically active" OR "older adults" OR "elderly") OR AB ("healthy" OR "adults" OR "adult" OR "subjects" OR "men" OR "women" OR athlete* OR "trained" OR "sedentary" OR "recreational" OR "non-athlete" OR "physically active" OR "older adults" OR "elderly") OR KW ("healthy" OR "adults" OR "adult" OR "subjects" OR "men" OR "women" OR athlete* OR "trained" OR "sedentary" OR "recreational" OR "non-athlete" OR "physically active" OR "older adults" OR "elderly") | 202,163 |
| 4 | TI ("randomized controlled trial" OR "controlled clinical trial" OR "randomized" OR "randomised" OR "randomly" OR "placebo" OR "trial" OR "RCT") OR AB ("randomized controlled trial" OR "controlled clinical trial" OR "randomized" OR "randomised" OR "randomly" OR "placebo" OR "trial" OR "RCT") OR KW ("randomized controlled trial" OR "controlled clinical trial" OR "randomized" OR "randomised" OR "randomly" OR "placebo" OR "trial" OR "RCT") | 42,070 |
| 5 | S1 AND S2 AND S3 AND S4 | 6 |
| CNKI（Date 2026-3-11） | | |
| 1 | (SU='植物乳杆菌' OR SU='Lactobacillus plantarum' OR SU='TWK10' OR SU='PS128') AND (SU='运动表现' OR SU='运动耐力' OR SU='肌肉力量' OR SU='爆发力' OR SU='运动恢复') AND (SU='随机对照试验' OR SU='随机' OR SU='安慰剂对照') | 0 |
| Wanfang Data（Date 2026-3-11） | | |
| 1 | 主题:("植物乳杆菌" + "Lactobacillus plantarum" + "TWK10" + "PS128") * 主题:("运动表现" + "耐力" + "肌肉力量" + "运动恢复") * 主题:("随机对照试验" + "随机" + "安慰剂对照") | 0 |
